# PTPROt aggravates inflammation by enhancing NF-κB activation in liver macrophages during nonalcoholic steatohepatitis

**DOI:** 10.7150/thno.42658

**Published:** 2020-04-06

**Authors:** Kangpeng Jin, Yang Liu, Yuze Shi, Haitian Zhang, YuanYuan Sun, Guangyan Zhangyuan, Fei Wang, Weiwei Yu, Jincheng Wang, Xuewen Tao, Xin Chen, Wenjie Zhang, Beicheng Sun

**Affiliations:** 1 Department of Hepatobiliary Surgery, Nanjing Drum Tower Hospital Clinical College of Nanjing Medical University, Nanjing, Jiangsu Province, P.R. China; 2 Department of Hepatobiliary Surgery, The Affiliated Drum Tower Hospital of Nanjing University Medical School, Nanjing, Jiangsu Province, P.R. China; 3 Medical School of Southeast University, Nanjing, Jiangsu Province, China, P.R.China

**Keywords:** Mitophagy, Inflammasome, Reactive oxygen species, Mitochondria, NLR family pyrin domain containing 3

## Abstract

**Rationale**: Inflammation plays a crucial role in the progression of nonalcoholic steatohepatitis (NASH). Protein tyrosine phosphatase receptor type O truncated isoform (PTPROt) is an integral membrane protein that has been identified in osteoclasts, macrophages, and B lymphocytes. However, its relationship between inflammation and NASH is largely unknown. Herein, we aimed to study the function of PTPROt in NASH progression.

**Methods**: We established a NASH mouse model in wild-type (WT), PTPRO knockout mice by western diet (WD) and methionine-choline-deficient diet (MCD). In addition, MCD-induced NASH model was established in BMT mice. Moreover, we determined the expression of PTPROt in liver macrophages in human subjects without steatosis, with simple steatosis, and with NASH to confirm the relationship between PTPROt and NASH. *In vitro* assays were also performed to study the molecular role of PTPROt in NASH progression.

**Results**: Human samples and animal model results illustrated that PTPROt is increased in liver macrophages during NASH progression and is positively correlated with the degree of NASH. Our animal model also showed that PTPROt in liver macrophages can enhance the activation of the NF-κB signaling pathway, which induces the transcription of genes involved in the inflammatory response. Moreover, PTPROt promotes the transcription of pro-oxidant genes and inhibits antioxidant and protective genes via increased activation of the NF-κB signaling pathway, thereby causing an increased level of reactive oxygen species (ROS) and damaged mitochondria. This triggers the NLRP3-IL1β axis and causes a heightened inflammatory response. Notably, PTPROt partially limits inflammation and ROS production by promoting mitophagy, which participates in a negative feedback loop in this model.

**Conclusions**: Our data strongly indicate that PTPROt plays a dual role in inflammation via the NF-κB signaling pathway in liver macrophages during NASH. Further studies are required to explore therapeutic strategies and prevention of this common liver disease through PTPROt.

## Introduction

Nonalcoholic fatty liver disease (NAFLD) is the hepatic consequence of metabolic syndrome, which is the most common cause of chronic liver disease in industrialized and developing nations 1-3. NAFLD can range from simple steatosis to steatosis with progressive liver inflammation and fibrosis 4, a condition referred to as nonalcoholic steatohepatitis (NASH), which is generally considered irreversible and increases the prevalence of hepatocellular carcinoma 5-8. However, the mechanism of NASH pathogenesis remains unresolved.

Since its discovery nearly 20 years ago, nuclear factor (NF)-κB has emerged as one of the most intensely studied eukaryotic transcription factors, chiefly because of its pleiotropic effects and its inducible pattern of expression that is subject to multi-level regulation^9^, ^10^. Even though some studies show that the NF-κB signaling pathway regulates inflammation in NASH progression 11, 12, the relationship is still unclear.

During NASH progression, mitochondria are destroyed by excessive β-oxidation, which leads to mitochondrial metabolism dysfunction and produces ROS which breakdowns intracellular components and triggers several signaling pathways 13-16. Previous studies have demonstrated that activation of the NF-κB signaling pathway induces ROS production via transcriptional regulation of related oxidant genes. In addition, some studies have shown that NF-κB activation regulates the inflammasome-proinflammatory cytokine axis in macrophages 17.

Interestingly, to clear damaged mitochondria and excessive ROS, mitophagy, a microautophagy process that targets damaged mitochondria, plays a critical role in NASH progression 18-21. Previous studies have demonstrated that mitophagy is essential for the maintenance of cellular energy homeostasis and plays an important role in many physiological and pathological processes, including innate immunity 22, 23, aging 24, 25, cancer 26, neurodegenerative diseases^25^, ^27^, and tissue injury^28^. Emerging evidence has also confirmed that mitophagy acts as a significant inhibitor in the progression of NASH 19, 29. Thus, NF-κB-regulation of the ROS-inflammasome-proinflammatory cytokine axis and mitophagy may play an important role in the development of NASH.

Protein tyrosine phosphatase receptor type O (PTPRO) is a phosphotyrosine phosphatase (PTP) receptor which is an integral membrane protein and has been identified in many parenchymal cells, including lung, liver, and breast 30-32. However, PTPROt, its truncated isoform, is expressed in osteoclasts, macrophages, and B lymphocytes 33, 34. We have previously demonstrated that PTPRO represses the development of NASH through the PI3K-Akt signaling pathway in hepatocytes 35. Meanwhile, we also found that PTPROt aggravates inflammation by activating the NF-κB signaling pathway in macrophages 36. Interestingly, our previous studies showed that PTPRO was decreased in hepatocytes whereas there was an increase in liver macrophages with NASH. These suggested a difference between the function of PTPRO in hepatocytes and liver macrophages during NASH progression. Thus, understanding the function of PTPROt in liver macrophages during NASH progression and the relationship between PTPROt, NF-κB, ROS, mitophagy, and the inflammasome-proinflammatory cytokine axis may attribute to understanding the mechanism of NASH pathogenesis and allow for an exploration into potential preventative and therapeutic strategies for NASH.

## Methods

### Patients

Human liver samples were obtained from random individuals who underwent liver biopsy or surgical resection at Nanjing Drum Hospital (Nanjing, China). The descriptions of characteristics of human samples have been provided in the **Table S1**. The tissues from liver biopsy were used to make section and from surgical resection were used to isolate liver macrophages. The normal tissues were collected from the patients who received hepatic haemangioma surgery (n = 24) from January 2019 to July 2019, and the steatotic liver samples were collected from the eligible patients who received bariatric surgery (n = 86) from March 2018 to July 2019. Steatotic liver samples from individuals meeting any of the following criteria were excluded from the study: excessive alcohol consumption (>140 g for men or >70 g for women, per week), drug abuse, or viral infection (for example, infection with hepatitis B virus or hepatitis C virus). Samples with Steatosis score of 0, Ballooning score of 0, Lobular inflammation score of 0 as No steatosis. Steatosis score of 1, Ballooning score of 1, Lobular inflammation score of 1 as simple steatosis. Steatosis score of 2-3, Ballooning score of 2-3, Lobular inflammation score of 2-3 as NASH. All procedures that involved human sample collection were approved by the tissue bank of The Affiliated Drum Tower Hospital, Nanjing University (Nanjing, China). Informed consent for gene expression analysis of tissue was obtained from each patient before surgery, and the study was approved by our institutional ethics committee.

### Gene knockout mice

The PTPRO^+/-^ C57BL/6 mice, gifts from Dr. Bixby, University of Miami, were maintained and bred in the Model Animal Research Center of Nanjing University.

Precisely, a number of 24 wild type (WT) and 24 PTPRO KO (PTPROt^-/-^) mice of 6-8 weeks old were used and underwent operation. Mice at end point were sacrificed for livers or primary liver macrophages and serum. Portions of liver tissues were stored in liquid nitrogen for mRNA and protein isolation, and fixed in 4% paraformaldehyde for paraffin embedding.

All mice received human care according to National Institutes of Health recommendations outlined in their Guide for the Care and Use of Laboratory Animals. All animal experiments were approved by the Nanjing Medical University Institutional Animal Care and Use Committee.

### NASH Mouse model

8 week-old Wild-type and PTPRO KO Mice were fed with a Normal diet or MCD diet (Dyets, Bethlehem, PA, USA; #518810) for 4 weeks or Western diet [WD: purchased from Dyets, Inc. Wuxi, China) for 16 weeks. A total of 6 mice were in each group. These mice were euthanized after feeding with MCD diet for 4 weeks or WD diet for 16 weeks and had been confirmed to develop the histopathological features of NASH, such as steatosis, inflammation, and fibrosis.

### Bone Marrow Transplantation (BMT) Mouse model

Donor bone marrow (BM) cells from Wild-type and PTPRO KO Mice were flushed from tibiae and femora in RPMI 1640 (BioWhittaker, Verviers, Belgium). After erythrocyte lysis using a buffered ammonium chloride solution, BM cells were resuspended in phosphatebuffered saline (PBS). After the isolation procedures, viability of BM cells was always more than 90% by trypan blue dye exclusion. C57BL/6 mice, used as recipients, received a total body irradiation (TBI) 16 hours before BMT. A 6- or 7-Gy single dose TBI was applied with a dose rate of 2.7 Gy/minute. Recipients then received a single intravenous (in a lateral tail vein) injection (300 mL) containing BM cells. No immunosuppressive agents were given.

### The Serum Biochemistry Assay

Serum samples from WT or PTPROt^-/-^ mice were collected in each group. TG, TC, ALT, AST, LDL and HDL were measured by chemical analyzer Hitachi 7600-10 in the Department of Clinical Laboratory in Nanjing Drum Hospital.

### H&E, Oil Red O Staining

H&E and Oil-red-O staining was executed on the basis of a standard protocol. Photographs were taken with the microscope (OLYMPUS, BX43F) and software (XV Image Processing, 3.15.0.15404), and average values of integrated optical density (IOD) were obtained by analyzing five random fields per slide using Image-Pro Plus software v. 5.0. Every index was detected a minimum of three times.

### Macrophage isolation

Liver macrophages were isolated from WT and PTPROt^-/--^ mice fed with or without MCD or WD using the collagenase liver perfusion system. Briefly, the liver was perfused with a digestion buffer which contained collagenase for 5 minutes, chopped finely and filtered through a 70µm mesh. After centrifugation at 50g for 5 minutes, the hepatocyte fraction was discarded. Non-parenchymal cell fractions were layered on the top of Percoll gradients composed of 25% and 50% Percoll layers carefully. The liver macrophages fraction was isolated from between the two different Percoll gradient layers after centrifugation at 2300rpm for 30 minutes. And the isolated liver macrophages were cultured with RPMI media containing 10% FBS for 1-2 days.

To isolate liver macrophages from human liver samples, 2-3g fresh liver specimens were mechanically disrupted and dissociated with scissors (in 1-2 ml of cold complete RPMI). Dissociated tissue pieces were transferred into a 70μm strainer (placed into a 60-mm dish with 1-2 ml of cold complete RPMI) and further dissociated with the back of a 3-ml syringe. Cell suspension was filtered through 70μm strainers. And the homogenate was spun down at 400g for 5 min at 4 °C. Pellet was resuspended in 33% Percoll solution containing 10 U/ml heparin, and centrifuged for 15 min at 500g at room temperature. Pellet was lysed with ammonium chloride potassium buffer. After incubation with Fc-blocker (2.4 G2; BD PharMingen, San Diego, CA), and stained with FITC-conjugated CD68 Ab with Cy5-streptavidin (ARG23069; Arigo biolaboratories Corp, Shanghai, China), the liver macrophages were sorted by Flow cytometry [FACS Aria II Cell Sorter (BD Biosciences)].

### Fluorescent-activated Cell Sorting (FACS)

APC-anti-F4/80 (17-4801-82; eBioscience), FITC-anti-CD11b (11-0112-82; eBioscience), PerCP-Cyanine5.5-anti-Ly-6C (45-5932-80; eBioscience) were used to stain marcophages ioslated from tissues. Then, FACS Aria II Cell Sorter (BD Biosciences) and FlowJo software (Tree Star) were used for FACS assay.

### Enzyme-Linked Immunosorbent Assay

ELISA was performed on tissue homogenate collecting from WT and PTPROt^-/-^ mice liver using the Mouse IL-1β; TNFɑ; IL-6; IL-8; IL-17; IL-18 Platinum ELISA kit (shown in **Table S2**) per the manufacturer's instructions. All plants were detected by Spectrophotometer (Tecan Austria GmbH Untersbergstr.1A, REF:30086376) and software (Tecan, C25313).

### Cell Culture and Treatment

All the cells were purchased from Shanghai Institute for Biological Science (China). RAW264.7 cells were cultured in DMEM and liver macrophages, the primary Kupffer cells and monocyte-derived macrophages from mice were cultured in RPMI supplemented with 10% FBS, 100 U/ml penicillin, and 100 μg/ml streptomycin (Invitrogen, CA, USA) at 37℃ in a humidified incubator with 5% CO_2_.

For cell treatment, the FFAs mixture was prepared with oleic acid (OA; Sigma, O1257) and palmitic acid (PA; Sigma, P0500-10G) in a 2:1 ratio and used at a 0.5 mmol/L final concentration, for specified time periods (0, 6, 12, 18, 24 hours) to stimulate excessive uptake of FFAs.

CCCP/FFAs and/or PDTC was prepared with relative powder in DMSO and then mixed with DMEM or RPMI medium (Final concentration: CCCP (10 µM).

LPS (Sigma; L6529) was also prepared with relative powder in DMSO and then mixed with RPMI medium (Final concentration: LPS (100 ng/ml).

### Ectopic Expression and Gene Silencing

For overexpression of PTPROt were subcloned into the lentiviral vector PCDH-CMV-puro. Recombinant lentiviruses were produced by co-transfecting 293T cells with the lentiviral expression plasmid and packaging plasmids by Lipofectmine 3000 transfection reagent (Thermo Scientific) according to the manufacturer's protocol. Lentivirus was purified by ultracentrifugation and filtered for use.

### Cell transfection

The transfection of lentivirus stably expressing-PTPROt to RAW264.7 cells were cultured in six-well dishes, and carried out using polybrene (10μg/ml) for 24 hours. Finally, RAW cells stably overexpressing PTPROt (RAW-PTPROt^+^) were obtained successfully.

### Mitochondrial Assays by Mitotracker Red (Mito-Red)

Cells grown on coverslips were stained with Mitotracker Red Kit (M22425, Invitrogen, China) (final concentration, 100 nM) for 12 minutes in 37℃incubator, washed with PBS for three times, 5 minutes/each time. The primary liver macrophages and the PTPROt^+^ cells were then analyzed under an inverted confocal fluorescent microscope (Leica Microsystems CMS Gmbh Ernst-Leitz-Str, 17-37) and software (Leica Application Suite X, 3.6.20104.0) .

### Mitochondrial Assays by MitoSOX Red (Mito-SOX)

Cells grown on coverslips were also stained with MitoSOX Red Kit (M36008, Invitrogen, China) (final concentration, 5µM) for 10 minutes in 37℃incubator, washed with PBS for three times, 5 minutes/each time. The primary liver macrophages and the PTPROt^+^ cells were then analyzed under an inverted confocal fluorescent microscope (Leica Microsystems CMS Gmbh Ernst-Leitz-Str, 17-37) and software (Leica Application Suite X, 3.6.20104.0) .

### Immunofluorescence

For immunofluorescence in cells, related primary liver macrophages or RAW264.7 cells grown on coverslips were treated with CCCP/ FFAs/ vehicle and/or PDTC. Then, cells were stained with Mito-Red for 15min at 37℃ or not and next fixed in 4% paraformaldehyde for 30min. After permeabilization and blocking, cells were stained with Anti-Phospho-p65, anti-Tomm20 or anti-GFP-LC3 followed by secondary Alexa488-conjugated anti-mouse second antibody. Nucleuses were counterstained with DAPI.

All images were captured using an inverted confocal fluorescent microscope (Leica Microsystems CMS GmbH, TCS SP8) and software (Leica Application Suite X, 3.6.20104.0).

### Transmission electron microscopy (TEM)

Tissues were fixed in 4% formaldehyde and 1% glutaraldehyde and then were processed for transmission electron microscopy by Hitachi H-500 electron microscope (FEI, USA).

### Western-bolt analysis

Proteins were extracted from tissues or cultured cells using RIPA containing phenylmethanesulfonyl fluoride (Beyotime, Jiangsu, China). Then Equal amounts of proteins samples (100 µg) were separated using 7.5%/12.5% sodium dodecyl sulfate-polyacrylamide gel electrophoresis and transferred to polyvinylidene fluoride membrane. After blocking by 5% BSA in TBST, membranes were incubated with primary polyclonal antibodies (**Table S2**). Secondary anti-rabbit and anti-mouse horseradish peroxidase-linked antibodies were purchased from Santa Cruz Biotechnology (CA, USA). Blots were developed using electrochemiluminescence (ECL) reagent (Millipore, Billerica, MA, USA). Equal amounts of protein loading in each lane were confirmed using β-actin antibody. ImageJ software (NIH Image, Bethesda, MD, USA) was used to quantify the integrated density of the band.

### RNA isolation and Real-time quantitative PCR (RT-qPCR)

Mice tissue samples and all cells were homogenized in TRIzol (Invitrogen,Grand Island, NY, USA), and total RNA was isolated according to the manufacturer's suggested protocol: 1 μg of RNA was used for cDNA synthesis. To determine the relative level of cDNA, real-time PCR analyses were performed using an Applied Biosystems 7300 Detection System (Applied Biosystems®, CA). The real-time PCR reaction was performed according to the protocol of the SYBR® Premix Ex Taq™ kit (Takara, DRR041). Duplicate runs of each sample were normalized to β-actin to determine relative expression levels. All Primers were described in **Table S3**.

### Statistics

Data are expressed as the mean ± SEM. Differences between 2 groups were compared using the Student's t test. Differences between multiple groups were compared using 1-way ANOVA followed by Bonferroni's multiple comparisons test. *, **, *** and ****, indicate statistical significance with P <0.05, P < 0.01, P < 0.001 and P < 0.0001, respectively. Spearman′s rank correlation coefficient analysis was applied to analyze the relationships between associated factors. Statistically non-significant results were labeled as n.s. where appropriate. All analyses were performed using GraphPad Prism 6 software.

## Results

### PTPROt deficiency in liver macrophages restricts liver inflammation during NASH

**Figure 1:**
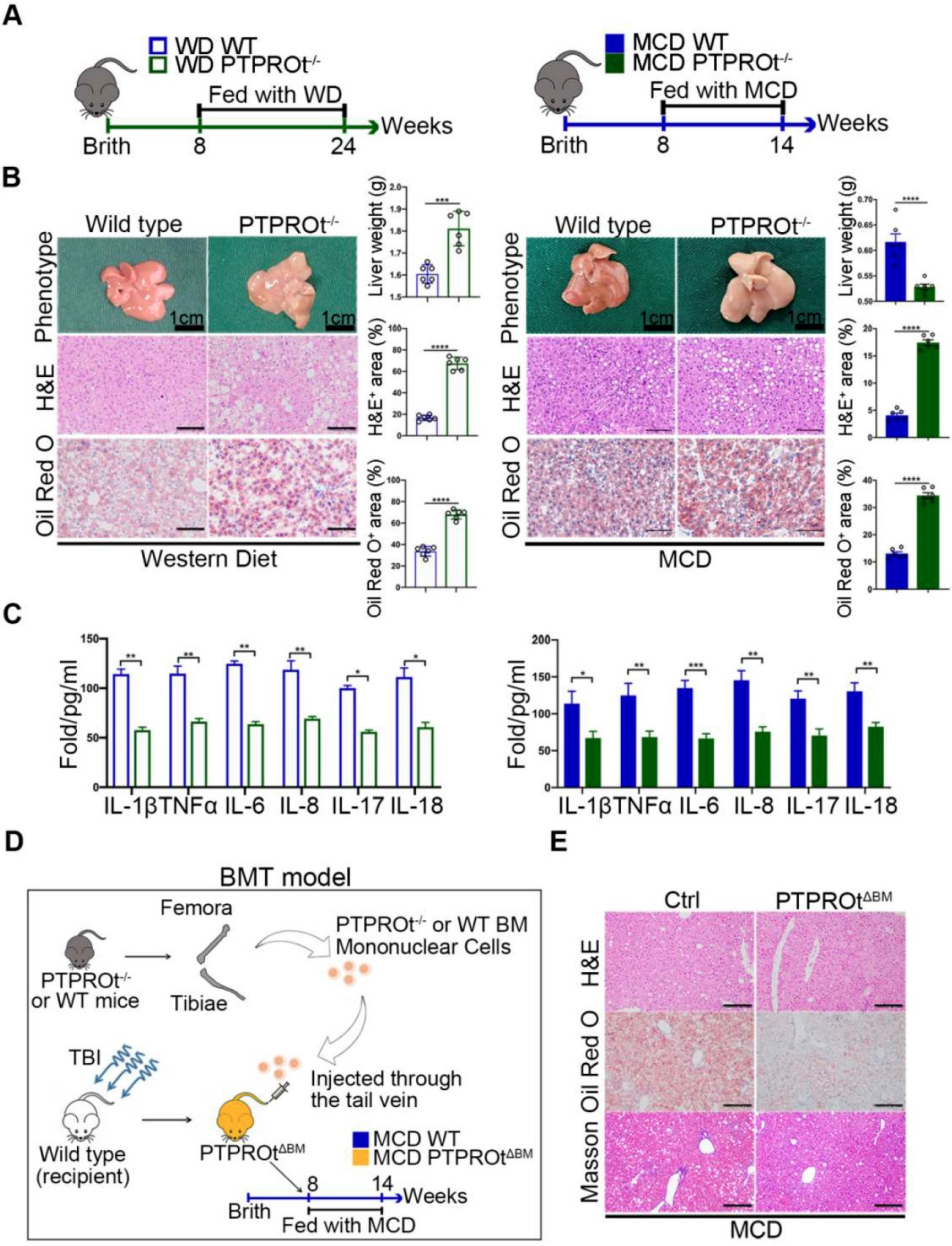
** Protein tyrosine phosphatase receptor type O truncated isoform (PTPROt) deficiency in liver macrophages causes decreased inflammation during nonalcoholic steatohepatitis (NASH). A.** The western diet-induced and methionine-choline-deficient (MCD)-induced NASH model in wild-type and PTPROt konckout mice. **B.**The liver from the mice described in Figure 1A (Bar = 1 cm). Liver sections were stained with Hematoxylin and Eosin (H&E) and Oil-Red O. Original magnification, ×20. Bar = 100 μm. Statistical analysis of liver weights and the number of H&E and Oil-Red O stained signal points in the above samples. **C.** The levels of IL-1β, TNFα, IL-6, IL-8, IL-17, and IL-18 in liver macrophages isolated from the liver samples in Figure 1B, as detected by ELISA. **D.** The MCD-induced NASH model in BMT mice. **E.** The liver from the mice described in Figure 1D. Liver sections were stained with Hematoxylin and Eosin (H&E), Oil-Red O and Masson. Original magnification, ×20. Bar = 100 μm. Abbreviations: BM: Bone marrow; BMT: Bone Marrow Transplantation; H&E: hematoxylin-eosin staining; MCD: Methionine-choline-deficient; PTPROt^ΔBM^: Bone marrow mononuclear-specific knockout PTPROt mice; TBI: Total body irradiation; WD: Western diet.

To clarify the function of PTPROt during NASH, western diet (WD)-induced and methionine-choline-deficient (MCD)-induced NASH models in wild-type and PTPROt knockout mice were used **(Figure 1A)**. These showed similar results with the PTPRO deficiency leading to worse NASH, which was demonstrated by an increased body and liver weight, a pale large liver, an increased hepatocyte vacuolation, and steatosis in WD-induced NASH model and an decreased body and liver weight, a pale smaller liver, an increased hepatocyte vacuolation, and steatosis in MCD-induced NASH model **(Figure 1B and Table S4-5)**. Serum levels of TG, TC, ALT, AST and LDL were higher and HDL was lower in PTPROt knockout mice compared with control groups **(Figure S1A-E and Table S4-5)**. Interestingly, ELISA results showed that a PTPROt deletion in liver macrophages caused a decreased level of inflammatory cytokines, including IL-1β, TNFα, IL-6, IL-8, IL-17, and IL-18 **(Figure 1C)**. This result is different than PTPRO deficiency which caused aggravated NASH. Meanwhile, Real time qPCR (RT-qPCR) also showed a similar result **(Figure S2A-B)**.

Thus, we transplanted the bone marrow isolated from PTPRO KO mice into Wild type mice treat with total body irradiation (TBI) **(Figure 1D)**. This model, Bone Marrow Transplantation (BMT) mice model 37, is a specific knockout PTPROt in all macrophages, which can clarify the role of PTPROt in liver macrophages, exclude the function of PTPRO in hepatocytes. This BMT mice was fed with MCD and showed that PTPROt deficiency in liver macrophages could suppress NASH progression **(Figure 1E)**. These results confirm that PTPROt promotes inflammation in liver macrophages during NASH, a role that is different from its function in hepatocytes.

### PTPROt deficiency in liver macrophages suppresses the transcription of inflammatory factors by limiting the NF-κB signaling

**Figure 2:**
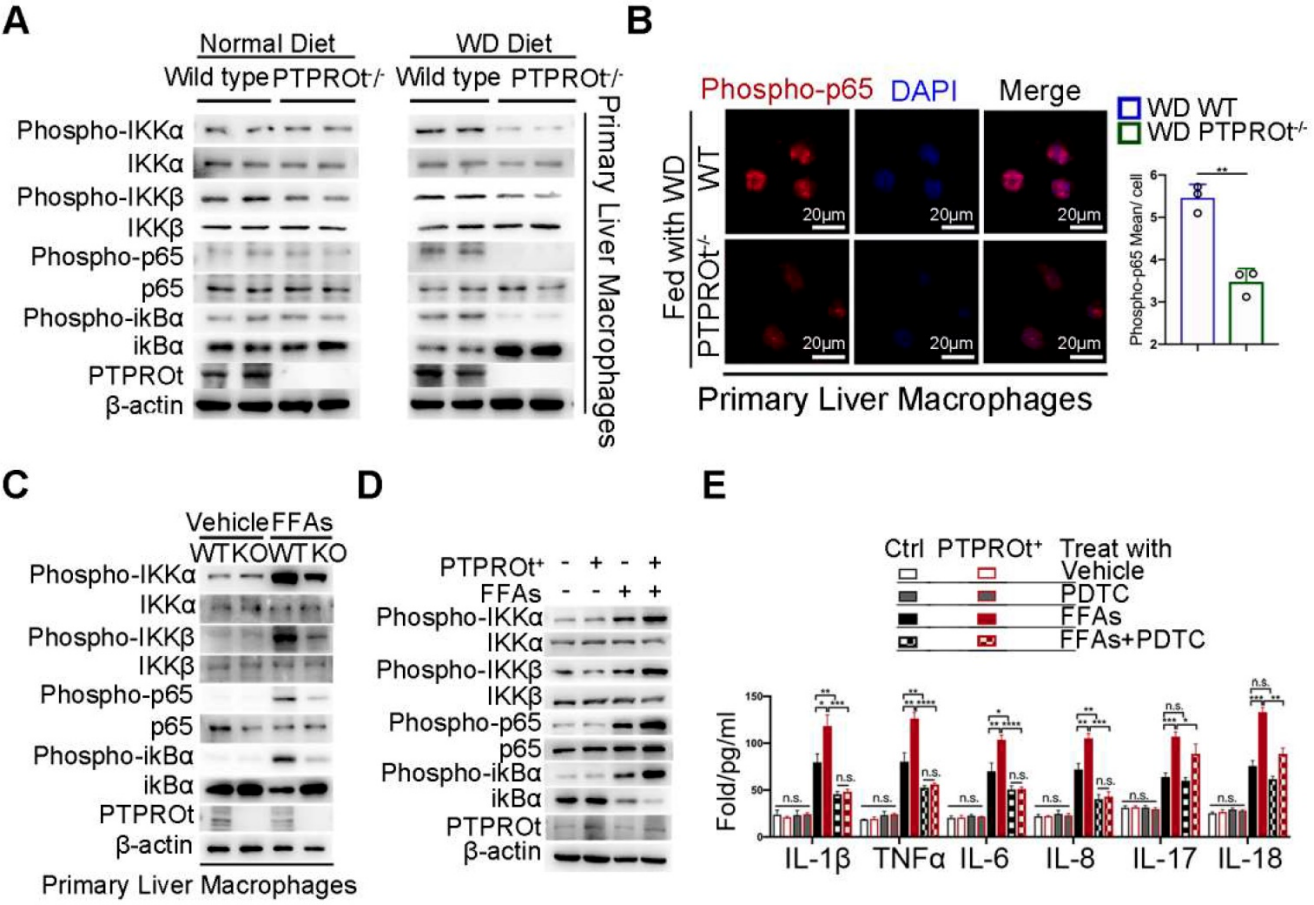
** PTPROt deficiency in liver macrophages causes decreased activation of the NF-κB signaling pathway. A.** Immunoblotting of phospho-IKKα, IKKα, phospho-IKKβ, IKKβ, phospho-P65, P65, phospho-ikBα, ikBα, PTPROt, and β-actin (loading control) in primary WT and PTPROt^-/-^ liver macrophages that were isolated from the mice described in **Figure 1A**.** B.** Fluorescence microscopy of phospho-p65 in primary WT and PTPROt^-/-^ liver macrophages isolated from the mice described in **Figure 1A**. DAPI, DNA-binding dye. Bar = 20 μm. Quantification of phospho-P65 per cell was shown.** C.** Immunoblotting of phospho-IKKα, IKKα, phospho-IKKβ, IKKβ, phospho-P65, P65, phospho-ikBα, ikBα, PTPROt, and β-actin (loading control) in primary liver macrophages isolated from WT and PTPROt^-/-^ mice treated with vehicle and free fatty acids (FFAs) for 24 hr.** D.** Immunoblotting of phospho-IKKα, IKKα, phospho-IKKβ, IKKβ, phospho-P65, P65, phospho-ikBα, ikBα, PTPROt, and β-actin (loading control) in RAW-ctrl and RAW-PTPROt^+^ cells treated with/without FFAs for 24 hr. E. The levels of IL-1β, TNFα, IL-6, IL-8, IL-17, and IL-18 in cell culture supernatants from RAW-ctrl and RAW-PTPROt^+^ cells treated with or without FFAs and/or PDTC for 24 hr were detected by ELISA. Abbreviations: DAPI: 4',6-diamidino-2-phenylindole; FFAs: Free Fatty Acids; KO: the primary liver macrophages isolated from PTPROt knockout mice; PDTC: Pyrrolidine dithiocarbamate; WT: the primary liver macrophages isolated from wild-type mice.

To reveal the mechanism of action for PTPROt, we harvested liver macrophages from the liver tissue in our NASH. Since PTPRO deficiency resulted in reduced NF-κB activation in hepatocytes and macrophages and this was correlated with c-Src phosphorylation, we examined the NF-κB signaling pathway. Immunoblot analysis showed that PTPROt deletion caused a decrease in the phosphorylation of proteins, including IKKα, IKKβ, ikBα, and p65 in liver macrophages from WD-induced and MCD-induced NASH models **(Figure 2A and Figure S3A)**. Liver macrophages comprise subsets of different cell populations, including liver-resident Kupffer cells (KCs) and monocyte-derived macrophages (MoMs)^38^. To clarify whether the function of PTPROt in these two subsets is similar, KCs and MoMs in our NASH model were isolated by FACS analysis that MoMs were defined as F4/80^+^ CD11b^hi^Ly6c^int^ and KCs were defined as F4/80^+^CD11b^lo^Ly6c^lo^ **(Figure S3B)**. Our immunoblot analysis also showed that PTPROt deletion caused a decreased NF-κB activation in these two subsets **(Figure S3C)**. These results demonstrated that PTPROt plays a similar role in KCs and MoMs. In addition, immunofluorescence showed a decreased level of nuclear phospho-p65 in liver macrophages with a PTPROt deletion compared with controls **(Figure 2B and Figure S3D)**, while PTPROt overexpression caused an increased level of nuclear phospho-p65 in liver macrophages **(Figure S3E).** These results also confirm the PTPROt function in NF-κB activation.

Meanwhile, we harvested liver macrophages from the PTPROt^-/-^ and wild-type mice and used a lentivirus to overexpress PTPROt in RAW cells (RAW-PTPTOt^+^). Subsequently, a NASH environment was established in these cells by stimulating with free fatty acids (FFAs). Immunoblot analysis revealed that PTPROt deficiency caused a decreased activation of the NF-κB signaling pathway as a decreased level of phospho-IKKα, phospho-IKKβ, phospho-ikBa, and phospho-p65 **(Figure 2C)**, while PTPROt overexpression caused enhanced activation of the NF-κB signaling pathway since the same phosphorylated proteins were all increased **(Figure 2D)**. In order to clarify the role of PTPROt in NF-κB signaling pathway, the primary liver macrophages were treated with LPS. Immunoblot analysis revealed that PTPROt deficiency also caused a decreased activation of the NF-κB signaling pathway in liver macrophages under LPS treatment **(Figure S3F).**

Interestingly, pyrrolidine dithiocarbamate (PDTC), an inhibitor of the NF-κB signaling pathway, blocked PTPROt overexpression-induced enhancement of inflammatory gene transcription (IL-1β, TNFα, IL-6, and IL-8), but there was no change in IL-17 or IL-18. **(Figure 2E).** These results demonstrated that PTPROt deficiency in liver macrophages caused inhibition of the NF-κB signaling pathway, thereby suppressing proinflammatory cytokine levels.

### PTPROt deficiency in liver macrophages suppresses ROS production through NF-κB inhibition

**Figure 3:**
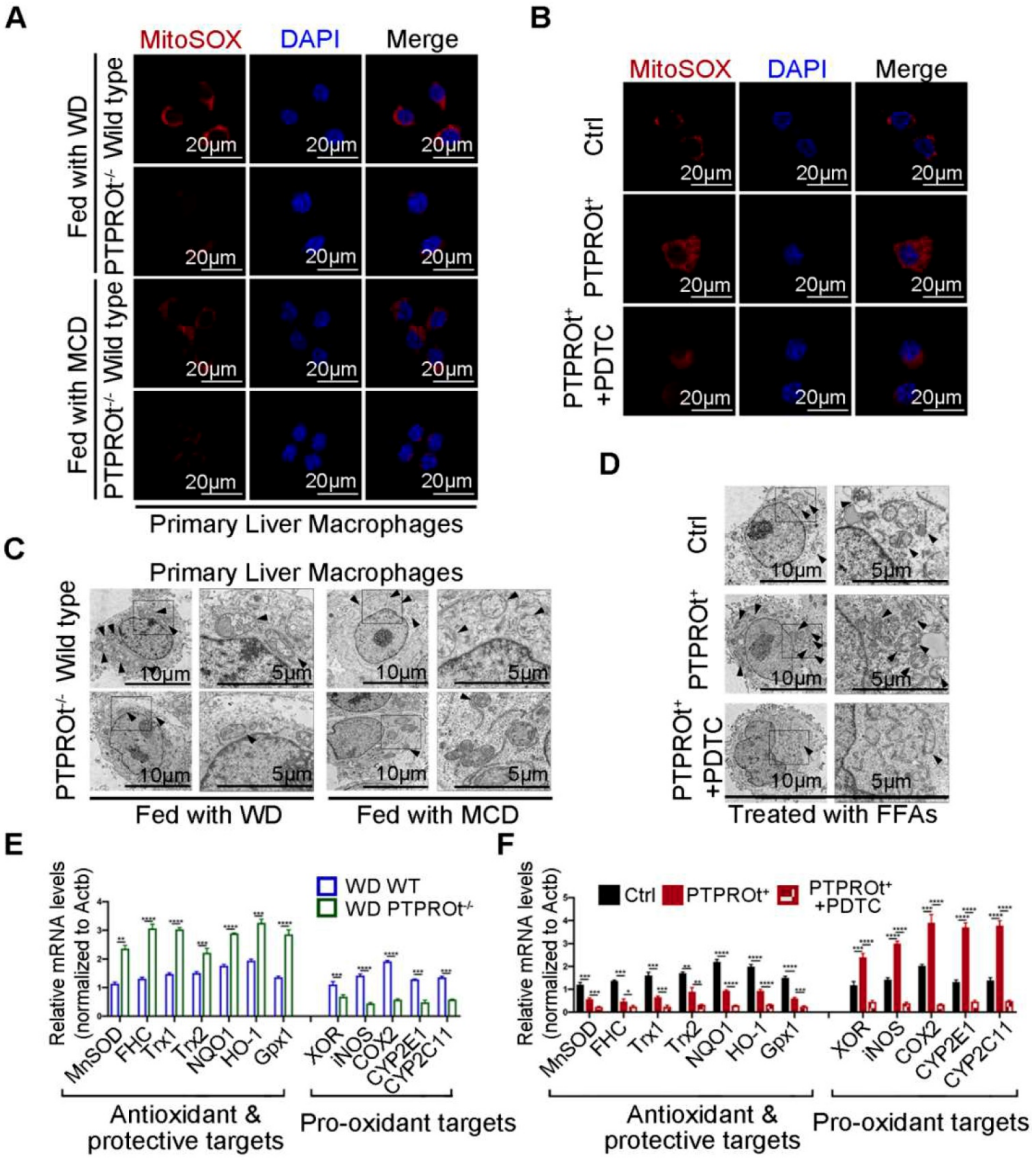
** PTPROt deficiency in liver macrophages suppresses the production of reactive oxygen species (ROS) by inhibiting NF-κB, which reduces pro-oxidant gene expression and promotes antioxidant gene expression. A.** Fluorescence microscopy of ROS (identified with MitoSOX) in primary liver macrophages isolated from the mice described in Figure 1A, B. DAPI, DNA-binding dye. Bar = 20 μm. B. Fluorescence microscopy of ROS (MitoSOX) in RAW-Ctrl or RAW-PTPROt^+^ cells treated with free fatty acids (FFAs), with or without pyrrolidine dithiocarbamate (PDTC) for 24 hr. DAPI, DNA-binding dye. Bar= 20 μm. **C.** Transmission electron microscopy of primary liver macrophages isolated from the mice described in Figure 1A, B. The arrows indicate mitochondria in liver marcophages. Bar (left) = 10 μm, Bar (right) = 5 μm. **D.** Transmission electron microscopy of RAW-Ctrl or RAW-PTPROt^+^ cells treated with FFAs, with or without PDTC for 24 hr. The arrows indicate mitochondria in liver marcophages. Bar (left) = 10 μm, Bar (right) = 5 μm. **E.** RT-qPCR results showing the relative mRNA levels of antioxidant and protective targets (MnSOD, FHC, Trx1, Trx2, NQO1, HO-1 and Gpx1) and pro-oxidant targets (XOR, iNOS, COX2, CYP2E1 and CYP2C11) in primary liver macrophages isolated from the mice described in Figure 1A, B. **F.** RT-qPCR results showing the relative mRNA levels of antioxidant & protective targets (MnSOD, FHC, Trx1, Trx2, NQO1, HO-1 and Gpx1) and pro-oxidant targets (XOR, iNOS, COX2, CYP2E1 and CYP2C11) in RAW-Ctrl and RAW-PTPROt^+^ cells treated with FFAs, with or without PDTC for 24 hr. Abbreviations: DAPI: 4',6-diamidino-2-phenylindole; FFAs: Free Fatty Acids; MCD: Methionine-choline-deficient; PDTC: Pyrrolidine dithiocarbamate; WD: Western diet; MitoSOX: Mitochondrial Superoxide Indicator.

Decreased levels of IL-1β, TNFα, IL-6, and IL-8 were caused by PTPROt deficiency-induced inhibition of NF-κB. However, changes in IL-17 and IL-18 are not linked to the NF-κB signaling pathway. Thus, further investigation was performed to understand the mechanism for PTPROt regulation of IL-17 and IL-18. ROS play a key role during NASH progression, not only in the activation of NF-κB, but also in mitochondrial dysfunction. Previous studies have shown that ROS production is related to IL-17 and IL-18. Thus, the ROS levels in liver macrophages isolated from the NASH model and RAW-PTPTOt^+^ cell lines treated with FFAs were determined by immunofluorescence. Results showed that PTPROt deletion caused a decreased level of ROS in liver macrophages **(Figure 3A and Figure S4A)**, while PTPROt overexpression increased ROS production **(Figure 3B and Figure S4B)**. Meanwhile, the condition of mitochondria in liver macrophages from the NASH model and RAW-PTPROt^+^ cells was determined by electron microscopy. Results showed that PTPROt deficiency leads to a decreased quantity of damaged mitochondria in liver macrophages **(Figure 3B and Figure S4C)**, while PTPROt overexpression caused more damaged mitochondria **(Figure 3D and Figure S4D)**.

Previous studies have shown that the NF-κB signaling pathway is closely associated with ROS, by influencing the transcription of pro-oxidant and antioxidant genes. Thus, we examined the expression of these genes in liver macrophages from our NASH mouse model. RT-qPCR showed that PTPROt deficiency caused decreased expression of pro-oxidant genes and increased expression of antioxidant genes **(Figure 3E and Figure S4E)**. Moreover, PTPROt overexpression in liver macrophages caused the opposite effect **(Figure 3F)**. In addition, inhibiting the NF-κB signaling pathway by treatment with PDTC blocked the effects induced by PTPROt overexpression **(Figure 3B, D, F, and Figure S4B, D)**. Taken together, these results revealed that PTPROt activates the NF-κB signaling pathway, promoting the transcription of antioxidant genes and reducing the transcription of pro-oxidant genes, thereby enhancing ROS production in macrophages.

### PTPROt deficiency causes decreased activation of the NLRP3-Caspase-1 axis via NF-κB inhibition

Previous reports have shown that ROS can damage mitochondria and activate the downstream inflammasome / inflammatory cytokine signaling pathway and PTPROt can regulate ROS production and mitochondrial degradation. When taken together, this suggests that PTPROt may regulate the inflammasome/inflammatory cytokine signaling pathway through NF-κB.

**Figure 4:**
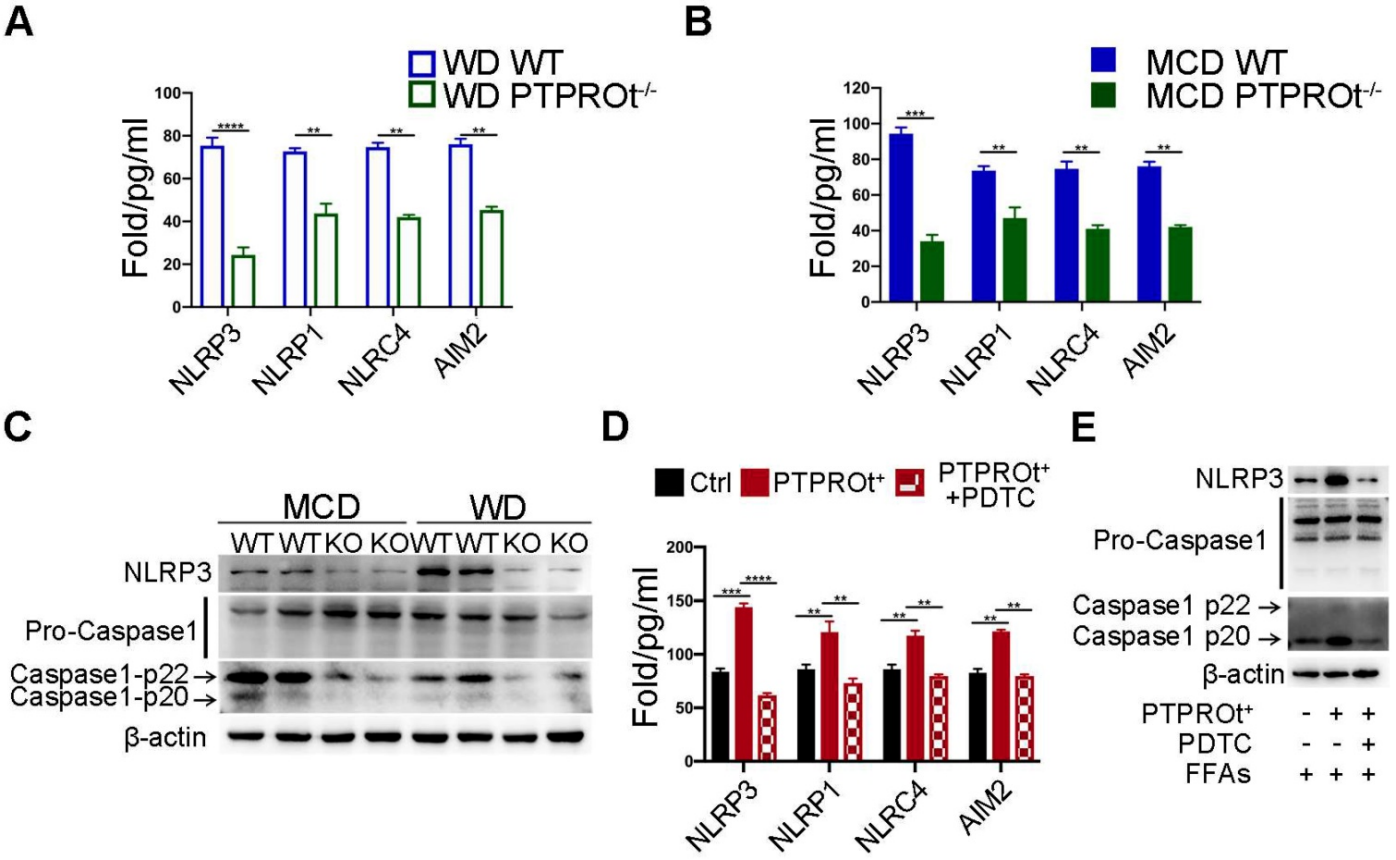
** PTPROt deficiency suppresses ROS production leading to decreased activation of the NLRP3-caspase-1 axis. A.** NLRP3, NLRP1, NLRC4, and AIM2 in cell homogenates from primary liver macrophages isolated from the mice described in **Figure 1A**. **B.** NLRP3, NLRP1, NLRC4, and AIM2 in cell homogenates from primary liver macrophages isolated from the mice described in **Figure 1B**. **C.** Immunoblotting of NLRP3, pro-caspase-1, caspase-1-p22, caspase-1-p20, and β-actin (loading control) in primary liver macrophages isolated from the mice described in **Figure 1A, B**. D. NLRP3, NLRP1, NLRC4, and AIM2 in cell culture supernatants from RAW-ctrl and RAW-PTPROt^+^ cells treated with free fatty acids (FFAs), with or without pyrrolidine dithiocarbamate (PDTC) for 24 hr. **E.** Immunoblotting of NLRP3, pro-caspase-1, caspase-1-p22, caspase-1-p20, and β-actin (loading control) in RAW-PTPROt^+^ cell lines treated with FFAs, with or without PDTC for 24 hr. Abbreviations: AIM2: Absent In Melanoma 2; MCD: methionine-choline-deficient diet; FFAs: Free fatty acids; NLRC4: NLR family CARD domain-containing protein 4; NLRP1: NLR family pyrin domain containing 1; NLRP3: NLR family pyrin domain containing 3; KO: the primary liver macrophages isolated from PTPROt knockout mice; PDTC: Pyrrolidine dithiocarbamate; WD: Western diet; WT: the primary liver macrophages isolated from wild-type mice.

Inflammasome levels were detected in liver macrophages from our NASH mouse model by ELISA. Results showed that PTPROt deficiency caused a decreased level of inflammasomes **(Figure 4A-B)**. Notably, the level of NLRP3 was markedly altered in the PTPROt deficiency groups **(Figure 4A-B)**. Immunoblot analysis also showed that PTPROt deficiency in liver macrophages caused a decreased level of NLRP3 and activation of caspase-1 in our NASH model **(Figure 4C)**. Additionally, PTPROt overexpression led to an increased level of inflammasomes, with an increased expression of NLRP3 and activation of caspase-1 **(Figure 4D)**. Moreover, inhibiting the NF-κB signaling pathway could completely block all these effects induced by PTPROt in liver macrophages **(Figure 4D-E)**. These results show that PTPROt deficiency suppresses ROS production leading to decreased activation of the NLRP3-caspase-1 axis.

### PTPROt deficiency suppresses mitophagy which partly limits inflammation during NASH

**Figure 5:**
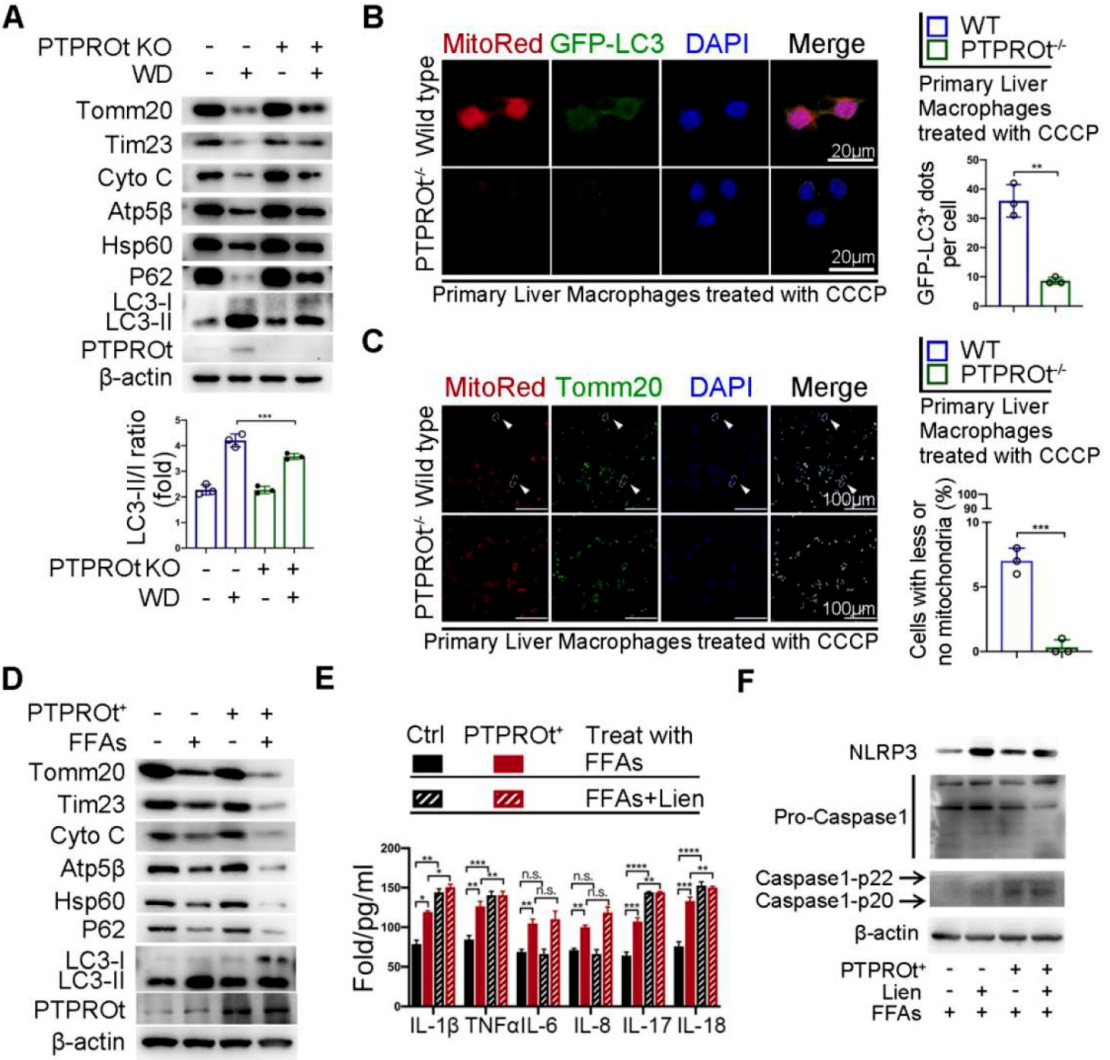
** PTPROt deficiency in liver macrophages aggravates inflammation and ROS production by limiting the NF-κB signaling pathway, resulting in suppressed mitophagy. A.** Immunoblotting of Tomm20, Tim23, Cyto C, Atp5β, Hsp60, P62, LC3-I, LC3-II, PTPROt, and β-actin (loading control) in primary liver macrophages isolated from the mice described in Figure 1A.** B.** Fluorescence microscopy showing co-localization of GFP-LC3 with mitochondria [identified with the mitochondrial stain MitoTracker Deep Red (Mito-Red)] in primary liver macrophages isolated from the mice described in Figure 1A treated with CCCP for 2 hr. Bar = 20 μm. Quantification of GFP-LC3 puncta co-localized with mitochondria per cell was shown. **C.** Fluorescence microscopy showing co-localization of anti-Tomm20 with mitochondria [identified with the mitochondrial stain MitoTracker Deep Red (Mito-Red)] in primary liver macrophages isolated from the mice described in Figure 1A treated with CCCP for 2 hr. The arrows indicate the cells staining with MitoRed^+^ and Anti-Tomm20^-^. Bar = 100 μm. Frequency of cells with few or no mitochondria was shown. **D.** Immunoblotting of Tomm20, Tim23, Cyto C, Atp5β, Hsp60, P62, LC3-I, LC3-II, PTPROt, and β-actin (loading control) in RAW-Ctrl and RAW-PTPROt^+^ cells treated with FFAs for 24 hr. **E.** ELISA results showing the levels of IL-1β, TNFα, IL-6, IL-8, IL-17, and IL-18 in cell culture supernatants of RAW-Ctrl and RAW-PTPROt^+^ cells treated with or without free fatty acids (FFAs) and/or liensinine for 24 hr. **F.** Immunoblotting of NLRP3, pro-caspase-1, caspase-1-p22/p20, and β-actin (loading control) in RAW-Ctrl and RAW-PTPROt^+^ cells treated with CCCP and/or liensinine for 2 hr. Abbreviations: CCCP: Carbonyl cyanide m-chlorophenylhydrazone; Cyto C: Cytochrome C; DAPI: 4',6-diamidino-2-phenylindole; FFAs: Free Fatty Acids: Lien: Liensinine; MitoRed: Mitotracker Red probe; WD:Western diet.

Previous reports have shown that NF-κB has a dual role in regulating inflammation. On one hand, the activation of NF-κB induces the transcription of inflammatory factors. On the other hand, NF-κB activation promotes mitophagy, which limits ROS production and activates inflammasome by clearing damaged mitochondria. Thus, we examined the level of mitophagy in liver macrophages from our NASH mouse model. Immunoblot analysis showed that PTPROt deletion caused an accumulation of the mitochondria as seen by the increased levels of Tomm20, Tim23, Cytochrome C (Cyto C), Atp5b, and Hsp60, while there was an inhibition of autophagy as seen by the accumulation of p62 and decreased LC3-II/LC3-I ratio **(Figure 5A and Figure S5A)**. Immunofluorescence revealed that PTPROt deficiency caused a decreased level of mitophagy and suppressed mitochondrial clearance **(Figure 5B-C)**. Also, PTPROt overexpression could suppress mitophagy in RAW cells treated with FFAs and CCCP **(Figure 5D and Figure S5B-C)**. Notably, inhibiting the activation of the NF-κB signaling pathway could block PTPROt overexpression-induced enhancement of mitophagy **(Figure S5D-E).** Interestingly, inhibiting mitophagy by liensinine could further increase the PTPROt-induced levels of IL-1β, TNFα, IL-17, and IL-18, while there was no difference in IL-6 or IL-8 levels **(Figure 5E)**. Meanwhile, blocking mitophagy could further enhance the PTPROt-overexpression-induced NLRP3-caspase-1 axis activation **(Figure 5F)**. These results demonstrated that the increased activation induced by PTPROt can somewhat limit inflammation and ROS production via NF-κB-induced mitophagy.

### Increased liver macrophages PTPROt expression in human fatty livers is associated with NAFLD severity

**Figure 6:**
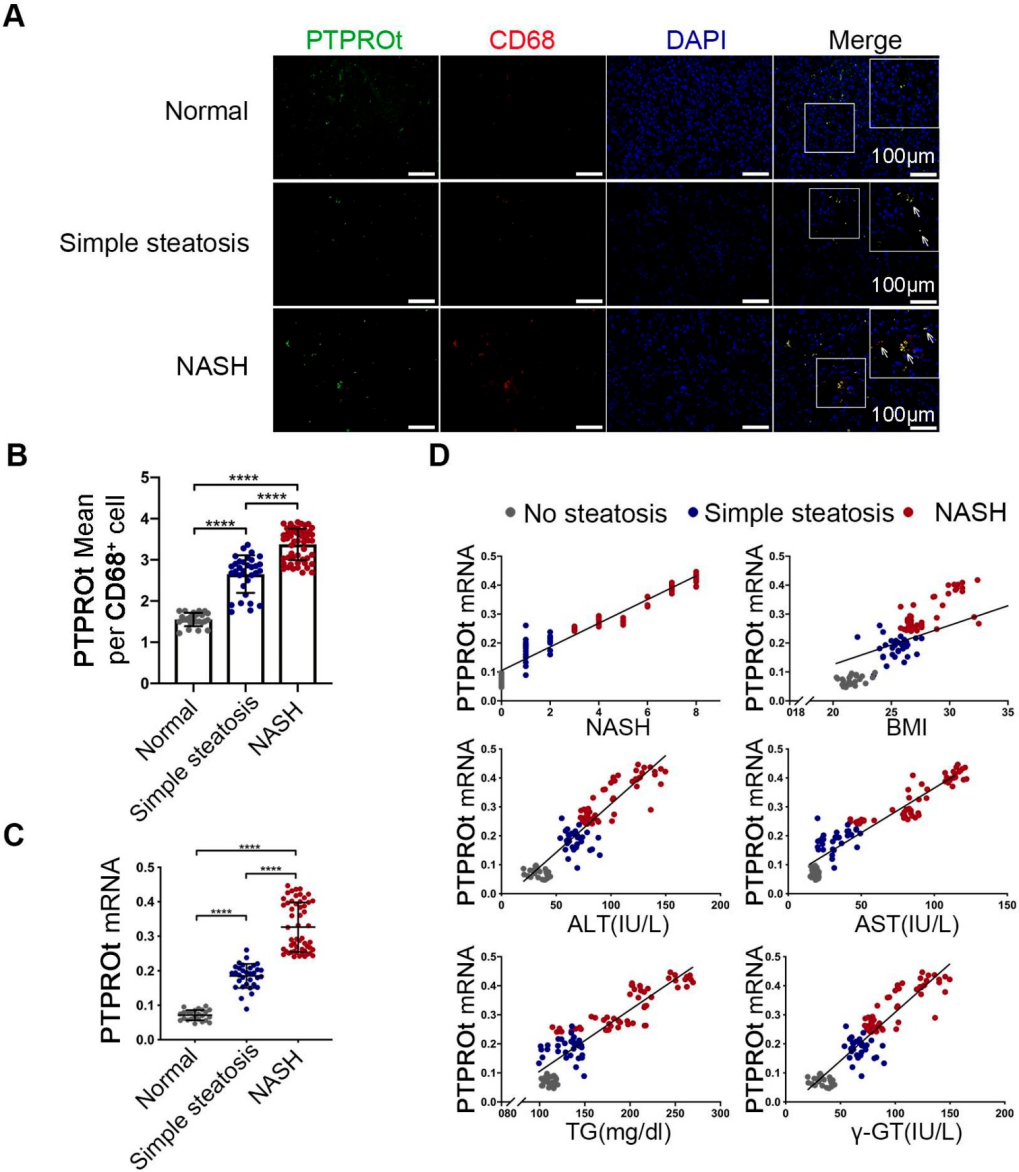
** Increased PTPROt expression in human fatty liver macrophages is associated with the severity of nonalcoholic fatty liver disease (NAFLD). A.** Fluorescence microscopy of PTPROt and CD68 in the section from the liver tissues of individuals without NAFLD (no steatosis; n = 24), with simple steatosis (n = 32), or with NASH (n = 54), described in Table S3. (Green: PTPROt; Red: CD68; Blue: DAPI; Bar = 100 μm). **B.** Statistical analysis of PTPROt signal points in Figure 6A. **C.** PTPROt mRNA levels in liver macrophages isolated from the livers of individuals without NAFLD (no steatosis; n = 24), with simple steatosis (n = 32), or with NASH (n = 54), described in Table S3. **D.** Pearson's comparison analyses of the correlation between PTPROt mRNA levels described in Fig 1.C and NASH (r = 0.9274), BMI (r = 0.6307), serum ALT concentrations (r = 0.8391), serum AST concentrations (r = 0.8739), serum TG concentrations (r = 0.7875) and serum γ-GT concentrations (r = 0.8391) (n = 110). P < 0.0001 for all of these correlations by Spearman's rank correlation coefficient analysis. Abbreviations: ALT: Alanine aminotransferase; AST: Alanine aminotransferase; BMI: body mass index; DAPI: 4',6-diamidino-2-phenylindole; HDL: High Density Lipoprotein; LDL: Low Density Lipoprotein; NASH: nonalcoholic steatohepatitis; PTPROt: Protein tyrosine phosphatase receptor type O truncated isoform; qRT-PCR: quantitative real-time PCR; TG: Triglyceride; γ-GT: γ-glutamyl transpeptidase.

To determine the clinical relevance of PTPROt in liver macrophages during NASH progression, the liver macrophages were isolated from human liver tissue samples that came from individuals without steatosis, with simple steatosis, and with NASH. RT-qPCR and immunofluorescence showed that mRNA and protein levels of PTPROt in liver macrophages were considerably higher in individuals with simple steatosis or NASH than in the nonsteatotic controls, and the NASH group had markedly higher PTPROt expression than the simple steatosis group **(Figure 6A-C)** (P<0.0001). Furthermore, PTPROt mRNA levels in liver macrophages were positively correlated with the degree of NASH, as seen in the activity score, body mass index (BMI), and biochemical criteria, including ALT, AST, TG and γ-GT **(Figure 6D and Table S1)**. These results demonstrate that PTPROt expression is increased in macrophages during human NASH progression and the expression is positively correlated with NASH.

## Discussion

NAFLD is the most popular chronic liver disease due to a westernized diet and sedentary lifestyle, which may cause the progression of NASH due to inflammation and fibrosis. However, the mechanism of NASH pathogenesis remains unresolved. This study showed that PTPROt, in liver macrophages, is a positive regulator of NASH progression.

PTPRO has been identified in many parenchymal cells, including lung, liver, and breast, whereas PTPROt, its truncated isoform, is expressed in osteoclasts, macrophages, and B lymphocytes 30-34, 39, 40. Previous studies have shown that PTPRO plays a dual role in hepatocytes and macrophages during ischemia-reperfusion injury 36. Moreover, PTPRO is a negative regulator of NASH in hepatocytes 35. Interestingly, our studies demonstrate that PTPROt expression is increased in liver macrophages during human NASH progression and is positively correlated with the degree of NASH. In addition, our NASH models confirm that PTPROt is a positive regulator of inflammation in liver macrophages during NASH. These results suggest the function of PTPROt in liver macrophages is different with it in hepatocytes during NASH. In addition, NASH in PTPRO knockout mice is suppressed while NASH in BMT mice is aggravated. These results demonstrate PTPRO in hepatocytes, as a suppressor, plays a major role during NASH progression, while PTPROt in liver marcophages, as an enhancer of inflammation, is a supplementary regulating mechanism which also plays a significant role in NASH.

NF-κB is a critical regulator of liver disease and can regulate a variety of pathological processes, such as inflammation 41, 42, cell proliferation, differentiation, and apoptosis 10, 43. A large number of studies have revealed that the activation of NF-κB leads to an increased level of inflammation. Consistent with this research, our study find that PTPROt caused an increased level of NF-κB activation, which promoted the transcription of proinflammatory cytokines, including IL-1β, TNFα, and IL-6. In addition, the levels of IL-17 and IL-18 were decreased in liver macrophages isolated from the PTPROt^-/-^ mouse NASH model. However, changes in IL-17 and IL-18 are not linked to the NF-κB signaling pathway. Our results clarify that PTPROt can promote IL-17 and IL-18 by triggering the inflammasome-proinflammatory cytokine axis (Caspase-1/NLRP3 etc.). Taken together, these results reveal that PTPROt aggravates inflammation via two ways, the NF-κB-induced transcription and the NF-κB-ROS-inflammasome-proinflammatory cytokine axis, in liver macrophages during NASH.

During NASH progression, mitochondria are damaged by excessive β-oxidation, which causes ROS production and destroys the intracellular environment 14, 15. Similarly, the present study demonstrates that PTPROt causes an increase in the number of damaged mitochondria and an increase in ROS production in liver macrophages via NF-κB activation. The affected mitochondria are cleared by mitophagy, a selective form of autophagy targeting damaged or excess mitochondria. Mitophagy is essential for the maintenance of cellular energy homeostasis and function 18-20. Previous studies have also demonstrated that NF-κB activation promotes mitophagy via increasing p62^44^. Interestingly, our study reveals that PTPROt can promote mitophagy, enhancing the clearance of damaged mitochondria and excessive ROS. Some reports have shown that mitophagy restrains the inflammasome-proinflammatory cytokine axis, including the NLRP3-IL1β axis and the AIM2-IL-18 axis, via damaged mitochondrial clearance and reduced ROS production, thus limiting inflammation during NASH 29, 45, 46. Similarly, our research reveals that an increased level of mitophagy induced by PTPROt suppresses the activation of the ROS-inflammasome-proinflammatory cytokine axis, thereby limiting inflammation in our NASH model. Notably, even though PTPROt promotes mitophagy to limit inflammation, this mechanism cannot completely mask the PTPROt-induced inflammation in liver macrophages. These results highlight the ability of PTPROt to serve as a positive regulator of inflammation in liver macrophages, while the PTPROt-NF-κB-mitophagy axis participates in a negative feedback loop for this mechanism at the same time.

Liver macrophages comprise subsets of different cell populations, including Kupffer cells and Infiltrating monocytes 38. Some reports have shown that in different stages of liver disease, resident Kupffer cells and freshly recruited monocyte-derived macrophages play a key part in the regulation of inflammation, fibrogenesis and fibrolysis^47^, and there are some difference between these two subsets^48^. However, our study reveals that PTPROt has a similar function in regulating NF-κB signaling pathway in KCs or MoMs during NASH progression. And some reports have also shown KCs and MoMs have a similar function in inflammation 48. Thus, we consider PTPROt as a promoter of inflammation during NASH, not only in Kupffer cells but in monocyte-derived macrophages.

Our study firstly identifies PTPROt as a positive regulator of inflammation in liver macrophages during NASH, even though PTPRO suppresses NASH progression in hepatocytes. Moreover, liver macrophage PTPROt plays a dual role in inflammation. During NASH progression, the major function of PTPROt is promoting inflammation via NF-κB-induced transcription and NF-κB-ROS-inflammasome-proinflammatory cytokine axis. As a supplement, PTPROt partially limits inflammation and ROS production by promoting mitophagy, which is part of a negative feedback loop in this model. Notably, PTPROt is increased in liver macrophages and is positively correlated with the degree of NASH during human NASH progression.

In summary, our observations emphasize the importance of PTPROt in liver macrophages for the progression of NASH. During NASH progression, PTPROt is increased in liver macrophages. On one hand, PTPROt can enhance the activation of the NF-κB signaling pathway, which induces the transcription of proinflammatory factors. On the other hand, PTPROt promotes the transcription of pro-oxidant genes and inhibits antioxidant and protective genes via enhanced activation of the NF-κB signaling pathway, thereby causing an increased level of ROS and damaged mitochondria, which trigger the NLRP3-IL1β axis, resulting in more inflammation. Notably, PTPROt partially limits inflammation and ROS production by promoting mitophagy, which plays a negative feedback role in this model.

## Conclusions

This study reveals that liver macrophages PTPROt plays a dual role in inflammation during NASH while providing insight into a novel molecular mechanism for NASH pathogenesis. These findings may help identify new strategies for preventing and treating human NASH.

## Supplementary Material

Supplementary figures and tables.

## Abbreviations

AIM2: Absent In Melanoma 2
ALT: Alanine aminotransferase
AST: Alanine aminotransferase
BM: Bone marrow
BMT: Bone Marrow Transplantation
BSA: Bovine Serum Albumin
CCCP: Carbonyl cyanide m-chlorophenylhydrazone
Cyto C: Cytochrome C
ELISA: Enzyme linked immunosorbent assay
FACs: Fluorescent-activated cell sorting
FFAs: Free fatty acids
HDL: High Density Lipoprotein
ikBα: Inhibitor of nuclear factor kappa-B kinase
IKKα: I-kappaB kinase alpha
IKKβ: I-kappaB kinase beta
IL-17: Interleukin-17
IL-18: Interleukin-18
IL1β: Interleukin-1 beta
IL-6: Interleukin-6
IL-8: Interleukin-8
IOD: Integrated optical density
KCs: Kupffer cells
KO: Knock out
LDL: Low Density Lipoprotein
Lien: Liensinine
LPS: Lipopolysaccharide
MCD: methionine-choline-deficient diet
Mito-Red: Mitotracker Red
MoMs: Monocyte-derived macrophages
NAFLD: Nonalcoholic fatty liver disease
NASH: nonalcoholic steatohepatitis
NF-κB: nuclear factor (NF)-κB
NLR: Family CARD Domain Containing 4
NLRP1: NLR family pyrin domain containing 1
NLRP3: NLR family pyrin domain containing 3
OA: Oleic acid
PA: Palmitic acid
PBS: Phosphatebuffered saline
PDTC: Pyrrolidine dithiocarbamate
PTP: phosphotyrosine phosphatase
PTPRO: Protein tyrosine phosphatase receptor type O
PTPROt^-/-^: PTPROt KO mice
PTPROt: Protein tyrosine phosphatase receptor type O truncated isoform
qRT-PCR: quantitative real-time PCR
ROS: reactive oxygen species
TBI: Total body irradiation
TC: Total cholesterol
TEM: Transmission electron microscopy
TG: Triglyceride
TNFα: Tumour necrosis factor alpha-like
WD: western diet
WT: wild type
γ-GT: γ-glutamyl transpeptidase
