# PTPROt aggravates inflammation by enhancing NF-κB activation in liver macrophages during nonalcoholic steatohepatitis: Erratum

**DOI:** 10.7150/thno.73372

**Published:** 2022-04-26

**Authors:** Kangpeng Jin, Yang Liu, Yuze Shi, Haitian Zhang, YuanYuan Sun, Guangyan Zhangyuan, Fei Wang, Weiwei Yu, Jincheng Wang, Xuewen Tao, Xin Chen, Wenjie Zhang, Beicheng Sun

**Affiliations:** 1 Department of Hepatobiliary Surgery, Nanjing Drum Tower Hospital Clinical College of Nanjing Medical University, Nanjing, Jiangsu Province, P.R. China; 2 Department of Hepatobiliary Surgery, The Affiliated Drum Tower Hospital of Nanjing University Medical School, Nanjing, Jiangsu Province, P.R. China; 3 Medical School of Southeast University, Nanjing, Jiangsu Province, China, P.R.China

In the original publication, errors were found in Figure 6D and Table S1. During statistics analysis, the Spearman analyze between PTPROt mRNA expression and γ-GT were misused with the data of ALT in Figure 6D and Table S1. The correct figures and table are shown below. The authors confirm that these corrections do not change the result interpretation or conclusions of the article. The authors are deeply sorry and sincerely apologize for any inconvenience or misunderstanding that may have caused.

## Figures and Tables

**Figure 6:**
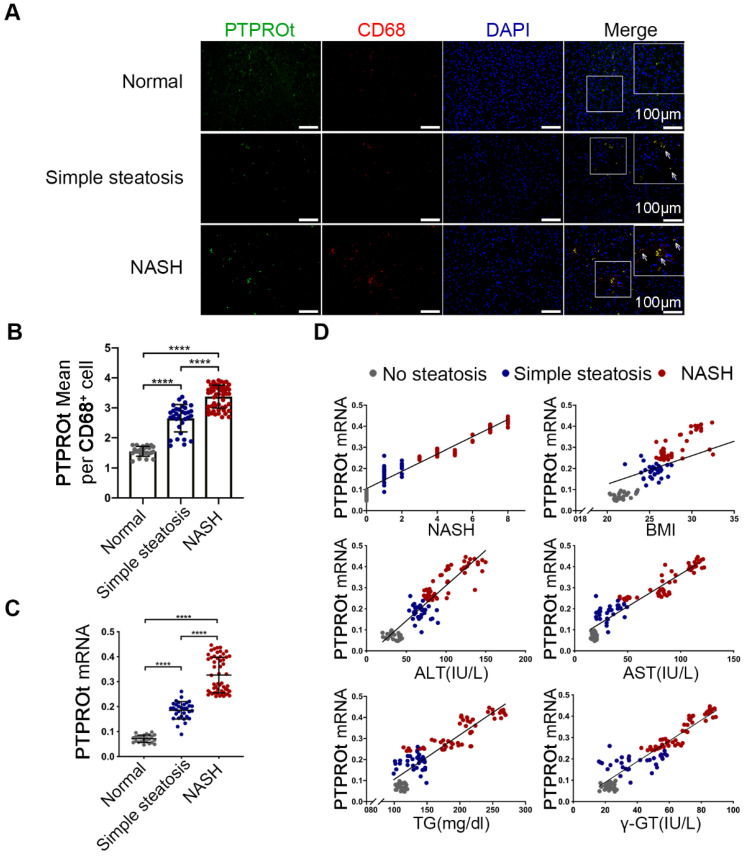
(Corrected figure) Increased PTPROt expression in human fatty liver macrophages is associated with the severity of nonalcoholic fatty liver disease (NAFLD). A. Fluorescence microscopy of PTPROt and CD68 in the section from the liver tissues of individuals without NAFLD (no steatosis; n = 24), with simple steatosis (n = 32), or with NASH (n = 54), described in Table S3. (Green: PTPROt; Red: CD68; Blue: DAPI; Bar = 100 μm). B. Statistical analysis of PTPROt signal points in Figure 6A. C. PTPROt mRNA levels in liver macrophages isolated from the livers of individuals without NAFLD (no steatosis; n = 24), with simple steatosis (n = 32), or with NASH (n = 54), described in Table S3. D. Pearson's comparison analyses of the correlation between PTPROt mRNA levels described in Fig 1.C and NASH (r = 0.9274), BMI (r = 0.6307), serum ALT concentrations (r = 0.8391), serum AST concentrations (r = 0.8739), serum TG concentrations (r = 0.7875) and serum γ-GT concentrations (r = 0.8624) (n = 110). P < 0.0001 for all of these correlations by Spearman's rank correlation coefficient analysis. Abbreviations: ALT: Alanine aminotransferase; AST: Alanine aminotransferase; BMI: body mass index; DAPI: 4',6-diamidino-2-phenylindole; HDL: High Density Lipoprotein; LDL: Low Density Lipoprotein; NASH: nonalcoholic steatohepatitis; PTPROt: Protein tyrosine phosphatase receptor type O truncated isoform; qRT-PCR: quantitative real-time PCR; TG: Triglyceride; γ-GT: γ-glutamyl transpeptidase.

**Table A:**
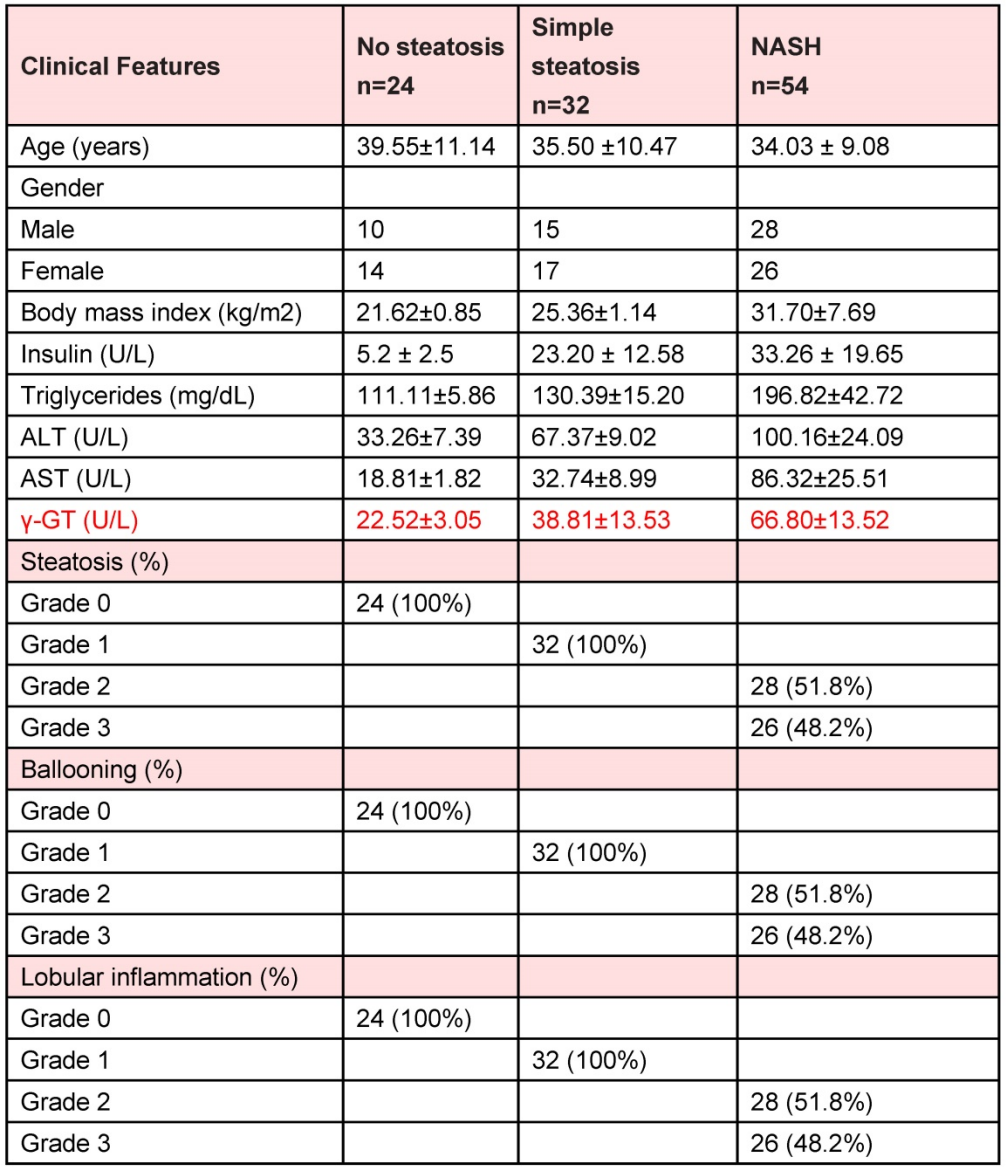
Corrected Table S1: The related characteristics of liver tissue samples of human subjects without steatosis, with simple steatosis and with NASH

