# Supplementary material for: PTPROt aggravates inflammation by enhancing NF-κB activation in liver macrophages during nonalcoholic steatohepatitis
Source: Theranostics. 2020 Apr 6;10(12):5290–304. doi: 10.7150/thno.42658 (PMC7196286; doi:10.7150/thno.42658)
Supplement: Supplementary file 1 — Supplementary figures and tables. [file thnov10p5290s1.pdf]

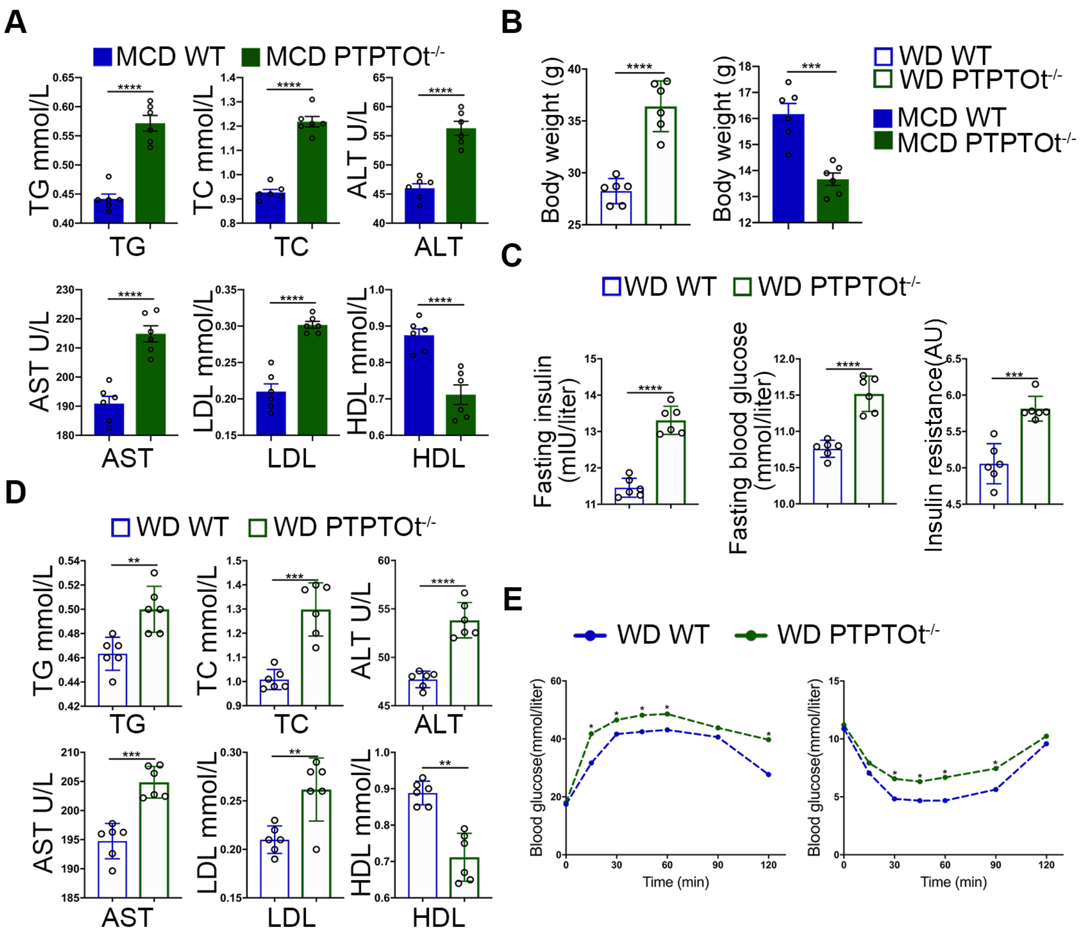

Figure S1

A. Statistical analysis of TG, TC, ALT, AST, LDL, and HDL in serum extracts from the mice described in **Figure 1B**.

B. Statistical analysis of body weight from the mice described in **Figure 1A, B**.

C. Statistical analysis of fasting insulin, fasting blood glucose, and insulin resistance from the mice described in **Figure 1A**.

D. Statistical analysis of TG, TC, ALT, AST, LDL, and HDL in serum extracts from the mice described in **Figure 1A**.

E. Glucose tolerance test (GTT) and insulin tolerance test (ITT) of mice described in **Figure 1A**.

Abbreviations: ALT: Alanine aminotransferase; AST: Alanine aminotransferase; DAPI: 4',6-diamidino-2-phenylindole; GTT: Glucose Tolerance Test; HDL: High Density Lipoprotein; ITT: Insulin Tolerance Test; LDL: Low Density Lipoprotein; NASH: nonalcoholic steatohepatitis; TC: Total cholesterol; TG: Triglyceride;

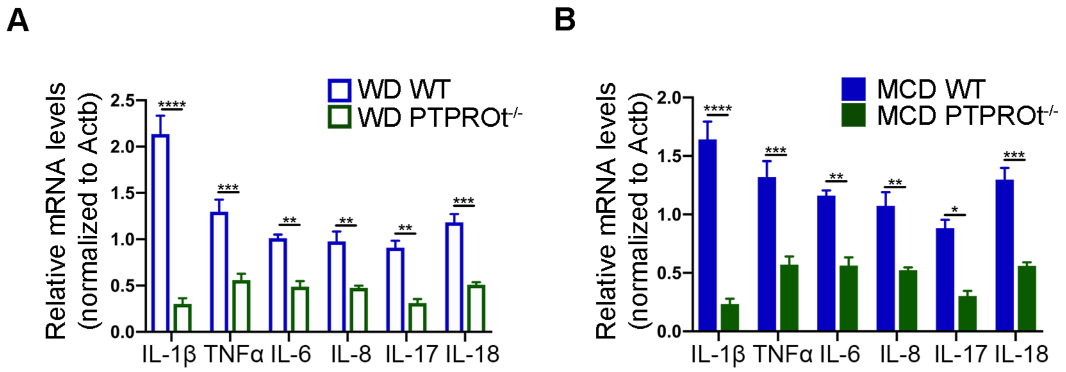

Figure S2

A. RT-qPCR results showing the mRNA levels of IL-1β, TNFα, IL-6, IL-8, IL-17, and IL-18 in liver macrophages isolated from the livers of mice described in **Figure 1A**.

B. RT-qPCR results showing the mRNA levels of IL-1β, TNFα, IL-6, IL-8, IL-17, and IL-18 in liver macrophages isolated from the livers of mice described in **Figure 1B**.

Abbreviations: MCD: Methionine-choline-deficient; WD: Western diet;

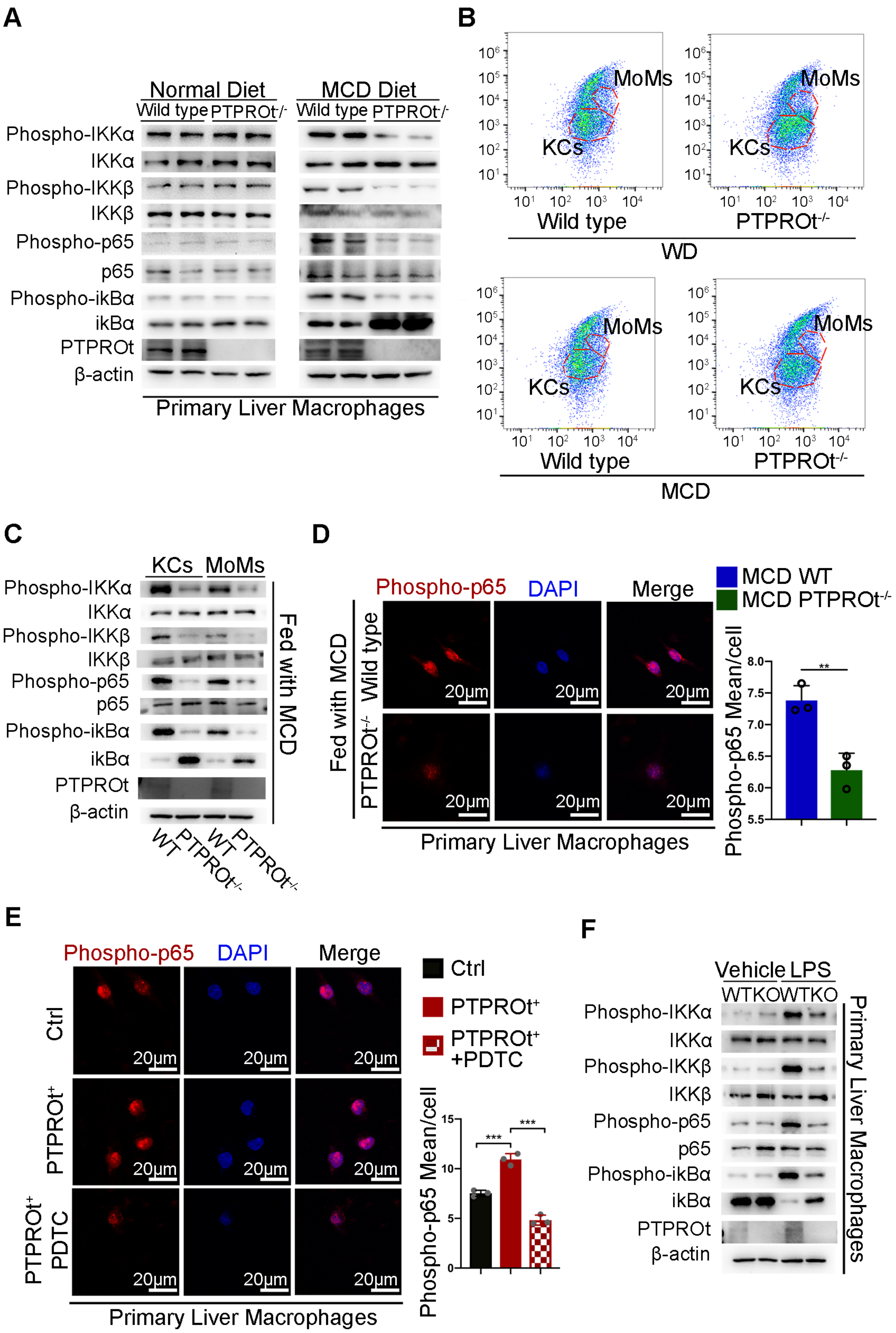

Figure S3

A. Immunoblotting of phospho-IKKα, IKKα, phospho-IKKβ, IKKβ, phospho-P65,

P65, phospho-ikBα, ikBα, PTPROt, and β-actin (loading control) in primary WT and PTPROt$^{-/-}$ liver macrophages that were isolated from the MCD-induced NASH model.

B. FACs analysis of monocyte-derived macrophages (MoMs) (defined as F4/80^{+}CD11b^{hi}Ly6c^{int}) and liver-resident Kupffer cells (KCs) (defined as F4/80^{+}CD11b^{lo}Ly6c^{lo}) from WD-induced and MCD-induced NASH model described in **Figure 1A**.

C. Immunoblotting of phospho-IKKα, IKKα, phospho-IKKβ, IKKβ, phospho-P65, P65, phospho-ikBα, ikBα, PTPROt, and β-actin (loading control) in primary WT/ PTPROt$^{-/-}$ monocyte-derived macrophages and Kupffer cells that were isolated from MCD-induced and WD-induced NASH model described in **Figure 1A.**

D. Fluorescence microscopy of phospho-P65 in primary WT and PTPROt$^{-/-}$ liver macrophages isolated from the mice described in MCD-induced NASH model. DAPI, DNA-binding dye. Bar = 20 μm. Quantification of phospho-P65 per cell was shown.

E. Fluorescence microscopy of phospho-p65 in RAW-Ctrl and RAW-PTPROt^{+} cells treated with free fatty acids (FFAs), with or without pyrrolidine dithiocarbamate (PDTC) for 24 hr. DAPI, DNA-binding dye. Bar = 20 μm. Quantification of phospho-P65 per cell was shown.

F. Immunoblotting of phospho-IKKα, IKKα, phospho-IKKβ, IKKβ, phospho-P65, P65, phospho-ikBα, ikBα, PTPROt, and β-actin (loading control) in in primary WT and PTPROt$^{-/-}$ liver macrophages treated with Vehicle/LPS.

Abbreviations: FFAs: Free Fatty Acids; KCs: Kupffer cells; MCD: Methionine-choline-deficient; LPS: Lipopolysaccharide; MoMs: monocyte-derived macrophages; PDTC: pyrrolidine dithiocarbamate; WD: Western diet;

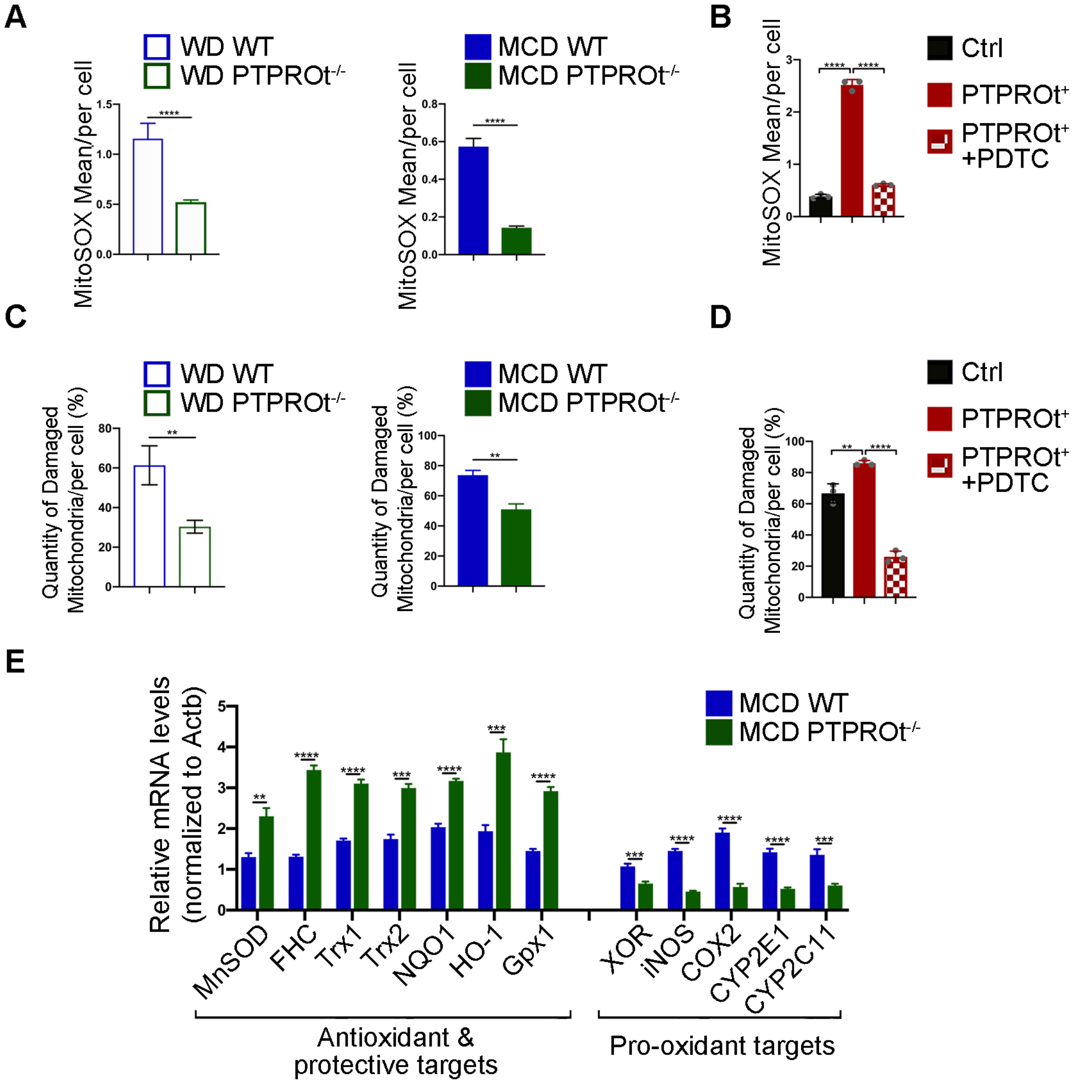

Figure S4

A. Corresponding to **Figure 3A**, statistical analysis of ROS levels in the liver macrophages of livers from the mice described in **Figure 1A, B**.

B. Corresponding to **Figure 3B**, statistical analysis of ROS level of RAW-Ctrl and RAW-PTPROt^{+} cells treated with FFAs, with or without pyrrolidine dithiocarbamate (PDTC) for 24 hr.

C. Corresponding to **Figure 3C**, the number of damaged mitochondria is shown.

D. Corresponding to **Figure 3D**, the number of damaged mitochondria is shown.

E. RT-PCR results showing the relative mRNA levels of antioxidant & protective targets (MnSOD, FHC, Trx1, Trx2, NQO1, HO-1and Gpx1) and

pro-oxidant targets (XOR, iNOS, COX2, CYP2E1, and CYP2C11) in primary liver macrophages from mice described in **Figure 1B**.

Abbreviations: MCD: Methionine-choline-deficient; WD: Western diet; PDTC: pyrrolidine dithiocarbamate;

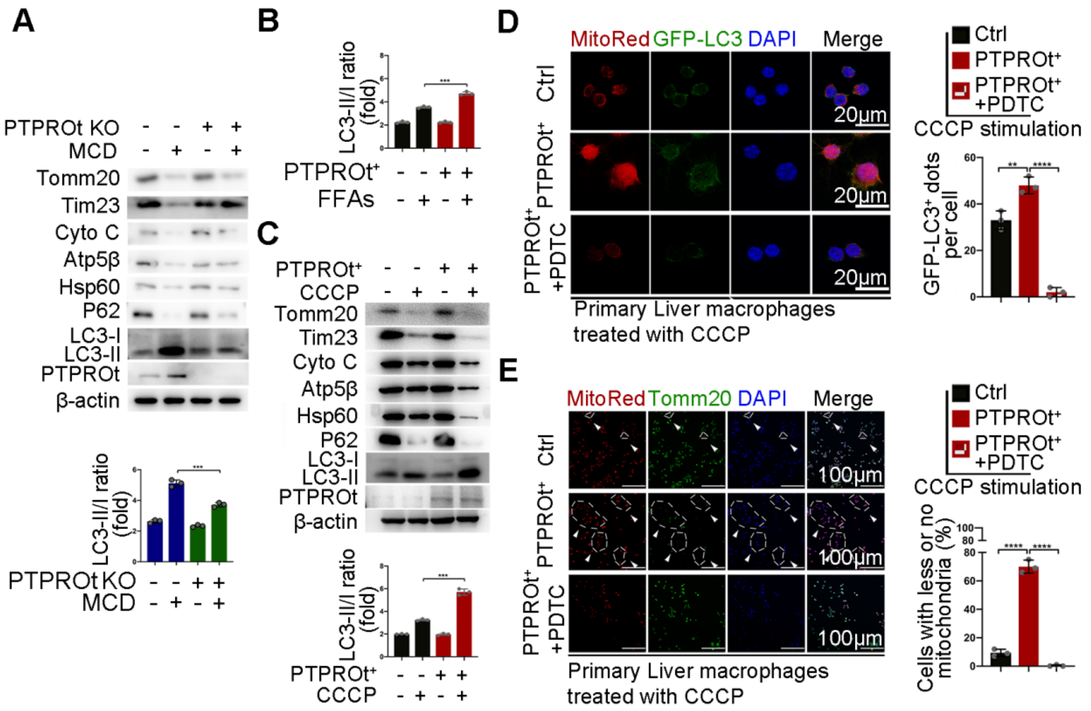

Figure S5

A. Immunoblotting of Tomm20, Tim23, Cyto C, Atp5β, Hsp60, P62, LC3I, LC3II, PTPROt, and β-actin (loading control) in primary liver macrophages isolated from the mice described in **Figure 1A**. LC3II/LC3I ratios were quantified using ImageJ software.

B. After densitometric analysis of blots corresponding to all samples in **Figure 5D**, LC3II/LC3I ratios were quantified using ImageJ software.

C. Immunoblotting of Tomm20, Tim23, Cyto C, Atp5β, Hsp60, P62, LC3I, LC3II, PTPROt, and β-actin (loading control) in RAW-Ctrl and RAW-PTPROt^{+} cells treated with CCCP for 2 hr. LC3II/LC3I ratios were quantified using ImageJ software.

D. Fluorescence microscopy showing co-localization of GFP-LC3 with mitochondria [identified with the mitochondrial stain MitoTracker Deep Red

(Mito-Red)] in RAW-Ctrl and RAW-PTPROt$^+$ cells treated with CCCP, with or without PDTC for 2 hr. Quantification of GFP-LC3 puncta co-localized with mitochondria per cell was shown.

E. Fluorescence microscopy showing co-localization of anti-Tomm20 with mitochondria [identified with the mitochondrial stain MitoTracker Deep Red (Mito-Red)] in RAW-Ctrl and RAW-PTPROt$^+$ cells treated with CCCP, with or without pyrrolidine dithiocarbamate (PDTC) for 2 hr. The arrows indicate the cells staining with MitoRed$^+$ and Anti-Tomm20$^-$. Frequency of cells with few or no mitochondria was shown.

Abbreviations: CCCP: Carbonyl cyanide m-chlorophenylhydrazone; Cyto C: Cytochrome C; DAPI: 4',6-diamidino-2-phenylindole; FFAs: Free Fatty Acids: MitoRed: Mitotracker Red probe; WD:Western diet;

Table S1: The related characteristics of liver tissue samples of human subjects without steatosis, with simple steatosis and with NASH

Clinical Features	No steatosis n=24	Simple steatosis n=32	NASH n=54
Age (years)	39.55±11.14	35.50 ±10.47	34.03 ± 9.08
Gender			
Male	10	15	28
Female	14	17	26
Body mass index (kg/m2)	21.62±0.85	25.36±1.14	31.70±7.69
Insulin (U/L)	5.2 ± 2.5	23.20 ± 12.58	33.26 ± 19.65
Triglycerides (mg/dL)	111.11±5.86	130.39±15.20	196.82±42.72
ALT (U/L)	33.26±7.39	67.37±9.02	100.16±24.09
AST (U/L)	18.81±1.82	32.74±8.99	86.32±25.51
γ-GT (U/L)	33.26±7.39	67.37±9.02	100.16±24.09
Steatosis (%)			
Grade 0	24 (100%)		
Grade 1		32 (100%)	
Grade 2			28 (51.8%)
Grade 3			26 (48.2%)
Ballooning (%)			
Grade 0	24 (100%)		

Grade 1		32 (100%)	
Grade 2			28 (51.8%)
Grade 3			26 (48.2%)
Lobular inflammation (%)			
Grade 0	24 (100%)		
Grade 1		32 (100%)	
Grade 2			28 (51.8%)
Grade 3			26 (48.2%)

Table S2

Antibody		
IKKα	CST	2628S
Phospho-IKKα	CST	2697S
IKKβ	abcam	ab55404
P65	CST	8242S
Phospho-P65	CST	3033S
ikBα	SANTA CRUZ	SC-847
Phospho-ikBα	SANTA CRUZ	SC8404
PTPRO	Proteintech	12161-1-AP
NLRP3	abcam	ab214185
Caspase-1-P20	Proteintech	22915-1-AP
Tomm20	CST	42406S
Tim23	BD BIOSCIENCES	611222
Cyto C	BD Biosciences	556433
Atp5β	SIGMA	HPA001520
HSP60	SIGMA	SAB4501464
P62	abcam	ab109012
LC3	CST	4108S
β-actin	Proteintech	66009
**Kit**		
Mouse IL-1 beta Platinum ELISA kit	R&D Systems, MLB00C	
Mouse TNFα Platinum ELISA kit	R&D Systems, MTA00B	
Mouse IL-6 Platinum ELISA kit	R&D Systems, M6000B	
Mouse IL-8 Platinum ELISA kit	R&D Systems, D8000C	
Mouse IL-17 Platinum ELISA kit	R&D Systems, M1700	
Mouse IL-18 Platinum ELISA kit	R&D Systems, 7625	
Mitotracker Red Kit	M22425, Invitrogen, China	

MitoSOX Kit	M36008, Invitrogen, China
**Drug**	
CCCP	#555-60-2; Sigma, Shanghai, China
PA	PA; Sigma, P0500-10G
OA	Sigma, O1257
PDTC	Sigma, P8765
Lien	#P0943; Pureone Bio Technology, Shanghai, China
LPS	Sigma, L6529

Table S3

Gene	Species	Forward Primer	Reverse Primer
IL-1β	Mus	GCAACTGTTCCTGAACTCAACT	ATCTTTTGGGGTCCGTCAACT
TNFa	Mus	CCCTCACACTCAGATCATCTTCT	GCTACGACGTGGGCTACAG
IL-6	Mus	CCAAGAGGTGAGTGCTTCCC	CTGTTGTTCAGACTCTCTCCCT
IL-8	Mus	CAAGGCTGGTCCATGCTCC	TGCTATCACTTCCTTTCTGTTGC
IL-17	Mus	TTTAACTCCCTTGGCGCAAAA	CTTTCCCTCCGCATTGACAC
IL18	Mus	CAAGGCTGGTCCATGCTCC	TGCTATCACTTCCTTTCTGTTGC
MnSOD	Mus	CAGACCTGCCTTACGACTATGG	CTCGGTGGCGTTGAGATTGT
FHC	Mus	CAAGTGCGCCAGAACTACCA	GCCACATCATCTCGGTCAAAA
Trx1	Mus	CATGCCGACCTTCCAGTTTTA	TTTCCTTGTTAGCACCGGAGA
Trx2	Mus	TGGGCTTCCCTCACCTCTAAG	CCTGGACGTTAAAGGTCGTCA
NQO1	Mus	AGGATGGGAGGTACTCGAATC	AGGCGTCCTTCCTTATATGCTA
HO-1	Mus	AAGCCGAGAATGCTGAGTTCA	GCCGTGTAGATATGGTACAAGGA
Gpx1	Mus	AGTCCACCGTGTATGCCTTCT	GAGACGCGACATTCTCAATGA
XOR	Mus	CACTGGGCAGACGAACTCTAC	TCTGGAAATAGGCCATACCCAT
iNOS	Mus	GTTCTCAGCCCAACAATACAAGA	GTGGACGGGTCGATGTCAC
COX2	Mus	TGAGCAACTATTCCAAACCAGC	GCACGTAGTCTTCGATCACTATC
CYP2E1	Mus	CGTTGCCTTGCTTGTCTGGA	AAGAAAGGAATTGGGAAAGGTCC
CYP2C11	Mus	GCGGCGTTCCTGAGTGTTTAT	CTGGCAGGTGGTTACCGTT
β-actin	Mus	GGCTGTATTCCCCTCCATCG	CCAGTTGGTAACAATGCCATGT
PTPRO	Ho	ACAAAACCCTTGCCTGTAACC	TTCCCCGAACTCCTCTTCTTT
β-actin	Ho	CATGTACGTTGCTATCCAGGC	CTCCTTAATGTCACGCACGAT

Table S4: 16 weeks WD-induced NASH mice model characteristics

	Wild type	*PTPROt KO*
Gender		
Male	6	6
Body weight /g	28.24±1.21	36.41±2.44
Liver weight /g	1.61±0.04	1.81±0.08
TG (mmol/L)	0.46±0.01	0.50±0.02
TC (mmol/L)	1.01±0.04	1.30±0.11
ALT (U/L)	47.72±0.85	53.83±1.83
AST (U/L)	194.76±3.03	204.87±2.71
LDL(mmol/L)	0.21±0.01	0.26±0.03
HDL(mmol/L)	0.89±0.03	0.71±0.07
Fasting Blood Glucose (mmol/L)	10.76±0.12	11.52±0.24
Insulin	11.46±0.26	13.31±0.39
Insulin Resistance (AU)	5.06±0.28	5.81±0.17
Ballooning (%)	16.67±2.58	67.50±5.75
Lipid accumulation (%)	33.67±4.50	68.00±4.29

Table S5: 4 weeks MCD-induced NASH mice model characteristics

	Wild type	*PTPROt KO*
Gender		
Male	6	6
Body weight /g	16.17±1.01	13.67±0.58
Liver weight /g	0.62±0.04	0.53±0.01
TG (mmol/L)	0.44±0.02	0.57±0.03
TC (mmol/L)	0.93±0.03	1.22±0.05
ALT (U/L)	46.00±1.92	56.30±2.94
AST (U/L)	190.88±6.14	214.85±6.76
LDL(mmol/L)	0.21±0.03	0.30±0.01
HDL(mmol/L)	0.88±0.04	0.71±0.07
Ballooning (%)	4.07±0.98	17.45±1.22
Lipid accumulation (%)	13.08±1.64	34.42±2.57
